# Synthesis of Gold Nanoparticles Using *Mimosa tenuiflora* Extract, Assessments of Cytotoxicity, Cellular Uptake, and Catalysis

**DOI:** 10.1186/s11671-019-3158-9

**Published:** 2019-10-26

**Authors:** Ericka Rodríguez-León, Blanca E. Rodríguez-Vázquez, Aarón Martínez-Higuera, César Rodríguez-Beas, Eduardo Larios-Rodríguez, Rosa E. Navarro, Ricardo López-Esparza, Ramón A. Iñiguez-Palomares

**Affiliations:** 1 Physics Department, University of Sonora, Rosales and Transversal, 83000 Hermosillo, Sonora Mexico; 2 Polymer and Material Department, University of Sonora, Rosales and Transversal, 83000 Hermosillo, Sonora Mexico; 3 Chemical Engineering and Metallurgy Department, University of Sonora, Rosales and Transversal, 83000 Hermosillo, Sonora Mexico

**Keywords:** Gold, Nanoparticles, Catalysis, Cellular uptake

## Abstract

Synthesis of gold nanoparticles (AuNPs) with plant extracts has gained great interest in the field of biomedicine due to its wide variety of health applications. In the present work, AuNPs were synthesized with *Mimosa tenuiflora* (Mt) bark extract at different metallic precursor concentrations. Mt extract was obtained by mixing the tree bark in ethanol-water. The antioxidant capacity of extract was evaluated using 2,2-diphenyl-1-picrylhydrazyl and total polyphenol assay. AuNPs were characterized by transmission electron microscopy, X-ray diffraction, UV-Vis and Fourier transform infrared spectroscopy, and X-ray photoelectron spectrometry for functional group determination onto their surface. AuMt (colloids formed by AuNPs and molecules of Mt) exhibit multiple shapes with sizes between 20 and 200 nm. AuMt were tested on methylene blue degradation in homogeneous catalysis adding sodium borohydride. The smallest NPs (AuMt1) have a degradation coefficient of 0.008/s and reach 50% degradation in 190*s*. Cell viability and cytotoxicity were evaluated in human umbilical vein endothelial cells (HUVEC), and a moderate cytotoxic effect at 24 and 48 h was found. However, toxicity does not behave in a dose-dependent manner. Cellular internalization of AuMt on HUVEC cells was analyzed by confocal laser scanning microscopy. For AuMt1, it can be observed that the material is dispersed into the cytoplasm, while in AuMt2, the material is concentrated in the nuclear periphery.

## Introduction

Plant-mediated biosynthesis of nanomaterials is an ecologically friendly method that allows NP synthesis in one-pot process. That is because the same bio-reducing agents of plant extracts act as stabilizing agents for the formed particles with a low rate of toxic compounds [1–3]. In this sense, *Mimosa tenuiflora* (Mt) bark has a high content of condensed tannins that have a structure of four flavonoid units [4], saponins, glucose, alkaloids (*N*,*N*-dimethyltryptamine), and starch [5–8]. These compounds (condensed tannins) can act as metal ion-reducing agents, but particularly, the flavonoid has been associated with metal complexation [4].

Mt ethanolic extract has been used as an antibacterial agent for gram negatives, gram positives, and yeast [9]. Also, as antiprotozoal (using flavonoids of leaves and flowers of Mt) [10] and on skin regeneration [6]. In addition, it has the potential to heal severe skin ulcers, whose properties were attributed to molecules and polyphenols of Mt bark [11]. Plant extracts show properties like antioxidants and particularly contain polyphenols that are used in green synthesis of metallic NPs such as Au, Ag, Fe, Pt, Pd, Cu, their alloys, and oxides [12, 13]. Optical properties of NP systems, as resonance frequency of surface plasmon resonance (SPR), are dependent of not only nanomaterials’ intrinsic characteristics (size, shape, dielectric constant), but also environment properties that surround to NPs, as the solvent where they are dispersed or nature of stabilizing molecules, that cover to NPs. These parameters are decisive to define the peak position of SPR in NP systems [14–16].

On the one hand, AuNPs catalytic properties have been reported in several works, related to organic compounds degradation such as pesticides, phenolic compounds, and dyes [17–19] and are used in catalytic processes related to environment remediation, for example, as cleaning contaminated water [20]. Several reports have emerged in recent years about AuNPs synthesized with plant extracts as black cardamom [21] and catalytic activity evaluations in dye degradation used in the industry, such as methylene blue (MB) [22], methyl orange [19], or Rhodamine B [23]. However, in the opposite direction, some papers have reported that NPs coated with stabilizing molecules showed poor catalytic activity due to sites available for catalysis on NP surface that was occupied by organic molecules [24, 25]. Also, AuNPs have been used as molecular sensors, such as colorimetric detection of toxic metal ions [7] and theragnostic (therapeutic and diagnostic) applications [26].

Functionalized AuNPs with molecules onto the surface show optical and biological properties associated with composition, thickness, organization, and conformation that define their features [27]. Nanotechnology has various challenges, such as aggregation of AuNPs on bloodstream [28]. AuNPs at concentration less of 20 μg/mL and with a size around 20 nm do not show cytotoxic effects in healthy and cancerous cell lines, and their use has allowed to analyze the interaction between NPs and cells [13, 29, 30]. Then, nanotechnology offers a possibility to interact at the same scale of cellular receptors [31] that allow to learn about cellular processes [32, 33] and antimicrobial properties [34], for example, in oxidative stress which generate a cascade of signaling for different effects, such as cytotoxicity or antioxidant defense response [35]. Further, functionalized AuNPs act as transporter vehicle of drugs, gene, or protein [36] and biomedical applications [37, 38]. Even more, AuNPs ligands as proteins and polymers generate a chemical environment that favors NPs internalization to reach the cytoplasm, nucleus, or keep out over the membrane [39].

In this work, AuNPs were synthesized using a rich polyphenolic Mt bark extract. AuMt cytotoxicity was evaluated in HUVEC cells, and cellular internalization was monitored by confocal microscopy at 24 h. AuMt catalytic activity on MB degradation, in the presence of sodium borohydride (NaBH_4_) at room temperature, was evaluated. Our results were compared with respect to similar works of catalysis with AuNPs synthesized by “green” methods.

## Materials and Methods

### Materials and Chemicals

For AuMt synthesis, 15 g of Mt tree bark was cut into pieces and placed in a 100-mL flask. Seventy milliliters of ethanol (Fermont, 99% pure) and 30 mL of ultrapure water (18 MΩ, Millipore) were added, then it was covered with aluminum and left at room temperature for 15 days. The solution was filtered using Whatman filter paper (8 μm) and later with an acrodisc (0.20 μm). The obtained solution was used as a reducing agent (Mt extract) for AuMt synthesis. A portion of the filtrate was rotoevaporated and then lyophilized for DPPH and total polyphenol assay and to construct a calibration curve of Mt extract. The concentration of Mt extract was 32.5 mg/mL, determined from a calibration curve. Tetrachloroauric acid (HAuCl_4_, Sigma-Aldrich 99% pure) was used as a metallic precursor. Concentrations of precursors used in synthesis were 5.3 mM for AuMt1 and 2.6 mM for AuMt2. The reducing agent volume was kept constant (1.6 mL), and the total volume sample was completed to 6 mL with ultrapure water. Additional file 1: Table S1 shows formulations used in AuMt1 and AuMt2 synthesis as well as the pH values for each reactant. The synthesis was performed at 25 °C under laboratory lighting conditions. The protocol used was as follows. In a 50-mL tube, the Mt extract solution is added followed by the ultrapure water, and finally, the gold precursor solution, stirring immediately in the vortex at 3000 rpm for 10 s. The synthesis of the AuMtNPs was visually confirmed within a few minutes by the change in coloration of the mixture. NPs clean process consists of centrifuging of suspension at 14,000 rpm for 1 h, discarding supernatant, adding water, and dispersing by sonication, repeating the process twice. After adding ethanol, AuMt are dispersed again by sonication and centrifuged at 14,000 rpm for 1 h. The supernatant is discarded and precipitated and is dried in an oven at a temperature of 40 °C. Then, the nanocomposite obtained is composed of AuNPs with Mt extract molecules on the surface.

### Time-Dependent pH Change of AuMtNP Synthesis

The pH of AuMtNP synthesis was measured as the reaction was carried out. For this, a multi-parameter pH/Conductivity Benchtop Meter (Orion™ VERSA STAR™) was used. The instrument was calibrated at 25 °C using a buffer reference standard solution for calibration at pH = 4.01. A recirculation bath was used to control the temperature of the samples at 25 °C (± 0.1 °C) in all measurements. pH was measured as the reaction was carried out for 180 s immediately after mixing the reagents. The same device was used in pH measurement of reactants.

### UV-Vis Spectra, 2,2-Diphenyl-1-Picrylhydrazyl (DPPH), and Total Polyphenol Assay

A double-beam Perkin-Elmer Lambda 40 UV/Vis spectrometer was used to obtain the extract’s UV-Vis spectrum, in a measurement range of 200–400 nm, with a scan rate of 240 nm/min. AuMt SPR was monitored between 250 and 875 nm.

AuMt formation kinetics was determined by measuring the absorbance at 550 nm every second while NP synthesis reaction was developed inside the quartz cell under magneto stirring.

For DPPH assays, all the tests were done by triplicate. Different Mt extract concentrations (25, 12.5, 6.25, and 3.125 μg/mL) were tested. One hundred microliters of ethanol was added to 100 μL of each concentration, in addition to the DPPH solution (300 μM). Subsequently, the samples were incubated for 2 h in the dark before measuring the absorbance at 517 nm. The results were compared with vitamin C and catechins (70 μmol/L), and both molecules were used as controls. For scavenging activity, DPPH radical dissolved on ethanol was used as a blank [40, 41]. The percentage of scavenging activity was computed with Eq. (1).

1 $$ \%\mathrm{Scavenging}\ \mathrm{activity}=\left[\left(1-\mathrm{A}\ \mathrm{sample}\right)/\mathrm{A}\ \mathrm{control}\right]\times 100 $$

where *A sample* is the sample absorbance and *A control* is the blank absorbance. Data were analyzed using analysis of variance (ANOVA) with Tukey multiple comparison tests.

For total polyphenol assay, the same concentrations were used by adding Folin-Ciocalteu at 0.25 N and sodium carbonate at 5% with a 1-h incubation in the absence of light. Absorbance was measured at 750 nm. The results are expressed as gallic acid equivalents [42, 43].

### Zeta Potential and DLS Size Determination

Zeta potential (ζ) of NPs was measured with Zetasizer NS (Malvern, PA), and sizes were measured by dynamic light scattering (DLS) of Zetasizer NS (resolution of 0.5 nm). The instrument calculates the ζ by determining the electrophoretic mobility (*μ*_*e*_) using Henry Eq. (2) [44]:

2 $$ {\mu}_e=\frac{2\varepsilon \zeta f(ka)}{3\eta } $$

where *ε*, *η*, and *f*(ka) denote the dielectric constant of the media, viscosity of media, and Henry’s function, respectively. Two values are generally used as approximations for the *f*(ka) determination, either 1.5 or 1.0. The electrophoretic determinations of ζ are the most commonly made in an aqueous solvent and moderate electrolyte concentration. *f*(ka), in this case, takes the value of 1.5 and is referred to as the classical Smoluchowski approximation, Eq. (3) [45].

3 $$ {\mu}_e=\varepsilon \frac{\upzeta}{\upeta} $$

The samples were placed into a U-shaped folded capillary cell for *ζ* measurements. Each sample was measured at room temperature (25 °C) in triplicate.

### Evaluation of NP Stability in Supplemented Culture Medium (s-DMEM)

AuMtNP stability was evaluated in s-DMEM by DLS and *ζ*. The hydrodynamic diameter (2R_H_) of AuMt1 and AuMt2 was measured at 37 °C in ultrapure water and s-DMEM at concentrations between 25 and 200 μg/mL. For AuMt1 and AuMt2 in s-DMEM, ζ was measured at 37 °C to establish if the culture media modifies the NP surface charge. Nanoparticles were added to an Eppendorf tube with s-DMEM previously thermalized and stirred in the vortex at 3000 rpm for 30 s. Incubation at 37 °C is kept for 15 min before taking the measurements at the same temperature.

### Fourier Transform Infrared Spectroscopy (FTIR)

Mt extract and the AuMt FTIR were obtained by a Perkin-Elmer Frontier FTIR using a solid sample. The spectrum was obtained on transmittance mode at a resolution of 2 cm^− 1^, from 4500 to 500 cm^−1^.

### X-ray Photoelectron Spectroscopy (XPS)

XPS experiment was carried out using a Perkin-Elmer (Model PHI 5100, resolution based on the FWHM of the Ag3d5/2 peak 0.80 eV, XR source dual-standard anode (Mg/Al), and 15 kV, 300 W, 20 mA). Survey scan analyses were carried out with a scan rate of 0.5 eV/s. For high-resolution analyses, a scan rate of 0.025 eV/s was used.

### Transmission Electron Microscopy (TEM)

For TEM, 10 μL of the sample was deposited on copper grids covered with a fomvar-carbon film (Electron Microscopy Sciences, 300 Mesh). Grids are left to dry for 1 h and placed in a vacuum chamber for 12 h. The electron microscopy equipment is a field emission Jeol 2010 F operated at 200 keV. Energy dispersive X-rays spectroscopy (EDS) is a detector Bruker Quantax 200, peltier cooled, and coupled to TEM system. Interplanar spacings of crystal planes revealed by high-resolution TEM (HRTEM) were determined by micrograph digital analysis (3.0 Gatan Version).

### X-ray Diffraction

Data were collected using a Bruker D8 QUEST diffractometer system, equipped with a multilayer mirror monochromator, and a CuKα Microfocus sealed tube (*λ* = 1.54178 Å). Frames were collected at *T* = 300 K via scans.

### AuMt Cytotoxic Effect

AuMt cytotoxic effect was evaluated in HUVEC cells using 3-(4,5-dimethylthiazolyl-2)-2,5-diphenyltetrazolium bromide (MTT) assay. Cells were grown on Dulbecco’s modified Eagle medium (DMEM, Sigma-Aldrich), supplemented with 10% fetal bovine serum (GibcoBRL) at 37 °C and 5% of CO_2_. HUVEC cells were counted in a Neubauer chamber, and viability was determined by trypan blue exclusion test (Sigma-Aldrich).

For MTT assay, cells were adjusted to 100,000 cells/mL, and 100 μL per well was placed in 96-well plates. AuMt1 and AuMt2 were evaluated at concentrations of 200, 100, 50, and 25 μg/mL. Treated cells were incubated for 24 and 48 h at 37 °C, 5% of CO_2_. After the incubation time, the plate was washed with phosphate-buffered saline (PBS) and MTT solution was added and incubated for 4 h. Dimethyl sulfoxide (DMSO) was added to disolve MTT crystals. Absorbance was measured at 570 nm on a multimode plate reader (Synergy HTX, BioTek), using the Gen5 software. Cell viability was computed using Eq. (4):

4 $$ \mathrm{Cell}\ \mathrm{viability}=\left(\mathrm{A}\ \mathrm{sample}/\mathrm{A}\ \mathrm{control}\right)\times 100\% $$

where *A sample* is the absorbance of the sample and *A control* is the absorbance of blank [46, 47].

### Statistical Analysis

Data are expressed as means ± standard deviations (SD). Significant differences between groups were analyzed by Tukey test, one-way ANOVA as appropriate. *P* values less than 0.05 were considered to be statistically significant. Origin Pro 9.1 software is used for data management, statistical analysis, and graph generation. The signs used are *p < 0.05. The performance with the treatment (AuMt1 and AuMt2) and the control group for 24 and 48 h was compared.

For live/dead assay, HUVEC cells were seeded on glass slides and treated with AuMt1 and AuMt2. After 24 h of incubation slides, these were stained using live/dead viability/cytotoxicity kit (ThermoFisher) under the manufacturer’s recommendation. The samples were observed by confocal laser scanning microscopy (CLSM800, Carl Zeiss).

### Confocal Laser Scanning Microscopy: Fluorescence of AuMt

Confocal microscopy analysis was carried out in a LSM 800 device (Carl Zeiss, Jena Germany) mounted on an inverted microscope Axio Observer.Z1 (Carl Zeiss, Jena Germany). Three lasers of 405, 488, and 640 nm, with a respective maximum power of 5, 10, and 5 mW, were used for the study. The fluorescence was collected using highly sensitive GaAsP detectors. Bright-field images were obtained by a collection of transmitted laser light on Photo Multiplier Tube (PMT). For AuMt fluorescence, live/dead assay, and NP distribution on HUVEC cell study, a Plan-Apochromatic × 40/0.95 dry objective was used. For 3D reconstruction cells with AuMt, a Plan-Aprochromatic × 63/1.40 oil objective was used.

For AuMt fluorescence characterization, a drop of 20 μL of NP colloidal dispersion was deposited in a cover glass and dried at room temperature before an analysis by CLSM. A 640-nm laser was employed as an excitation source at 0.5% of power, and the fluorescence was collected between 650 and 670 nm. Bright field AuMt images were formed using a 488-nm laser (0.2% of power) on transmitted light mode. The fluorescence and bright field were collected on separate tracks.

### Cellular Internalization

For AuMt internalization on HUVEC cells, the nucleus was stained with 4′,6-diamidino-2-fenilindol (DAPI) and the actin fibers with anti-β actin antibody coupled to fluorescein-5-isothiocynate (FITC) to delimit the cell border. DAPI was excited with a 405 nm laser at 1.0% of power and FITC with a 488-nm laser at 0.20%. Emissions of DAPI and anti-β actin antibody were collected between 410 and 500 nm and 500–700 nm, respectively. AuMt were excited with a 640 nm laser (0.50% power), and emission was collected between 650 and 700 nm.

3D cell-AuMt reconstructions and orthogonal projections were made from 30 images on Z-stack mode (total *Z* length = 8 μm), collecting fluorescence from DAPI, FITC, and AuMt as described above. Fluorescent signals were collected on separate tracks for each *Z* position. For clarity, the FITC signal was omitted on a 3D reconstruction.

A relative comparison of nanoparticle cellular uptake was realized. For this, the mean fluorescence intensity of AuMt1 and AuMt2 in HUVEC cells was determined from confocal images analysis using ImageJ software [48].

### Catalysis

Catalytic activity on MB, at a concentration of 3.33 × 10^−5^ M, was analyzed by UV-Vis spectroscopy. In homogeneous catalysis, 90 μL of NPs (2 mg/mL) was added directly in the quartz cell that contains MB and 200 μL of NaBH_4_ at a concentration of 100 mM. The sample was homogenized by magnet stirring inside of the spectrophotometer cell. The reaction was carried out at 25 °C.

## Results and Discussions

### Synthesis

By visual inspection, it was detected that NPs synthesis is very fast in both systems. The most intense color of AuMt1 system shown in the inset of Additional file 1: Figure S1 indicates a higher content of NPs from this synthesis. This is because AuMt1 has a double concentration of metallic precursor compared to AuMt2. In Additional file 1: Table S1, reagents used in nanoparticle synthesis have acidic pH. Additional file 1: Figure S1 shows the changes in pH of the reactions as AuMtNPs syntheses are carried out. Reactions start in an acidic environment (pH < 2.65), and as NPs synthesis develops, acidity grows. This is due to deprotonation of hydroxyl groups present in polyphenolic molecules of Mt extract. In fact, this is the first step of an oxide-reduction process that results in the transfer of electrons from deprotonated hydroxyl group to Au^3+^ ions. As products of oxide-reduction reaction, Au^3+^ ions are reduced to metal atoms Au^0^ and polyphenolic ring that contributes 2 electrons is oxidized. The process is described in the inset of Additional file 1: Figure S1.

#### UV-Vis Spectra, DPPH, and Total Polyphenol Assays

**Fig. 1:**
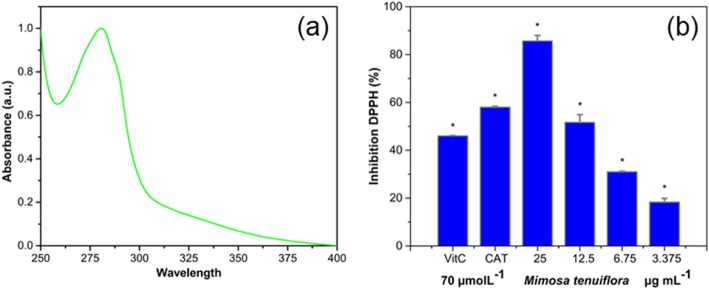
Characterization of Mt Extract. **a** Mt extract UV-Vis spectrum and **b** DPPH inhibition with one-way ANOVA analysis (*p < 0.05)

Mt bark extract UV-Vis spectrum is shown in Fig. 1a, where signal consists in a well-defined band with a maximum in 280 nm and broad of 50 nm. This spectrum is very similar to reported for *Rumex hymenosepalus* root extract, which has a high content of polyphenolic compounds [49]. Determinating the polyphenolic content in Mt bark extract is important because these molecules can contribute significantly as reducing agents in AuNPs synthesis, providing the necessary electrons for reduction of Au^3+^ ion to metallic gold (Au^0^). Once NPs are formed, polyphenolic compounds are absorbed on their surface providing stability to nanomaterials.

For DPPH assay, it was observed that for 12.5 mg/L of Mt extract, we obtained a 50% inhibition (L50), alike the values reported for Vitamin C and catechins (46 and 58%, respectively). This indicates that Mt extract possesses an antioxidant capacity very similar to pure compounds used as controls, Fig. 1b where significant differences (*p < 0.05) from control values are marked with an asterisk. The value of 425 mg/g obtained from total polyphenols assay indicates that almost half of the extracted mass is equivalent to gallic acid. The high antioxidant capacity and the high polyphenolic content in Mt extract suggest that it can be used as a good reducing and stabilizing agent nanomaterial synthesis within the framework of sustainable chemistry [50, 51].

### Characterization

#### Kinetic of Formation and UV-Vis Spectra AuMt

**Fig. 2:**
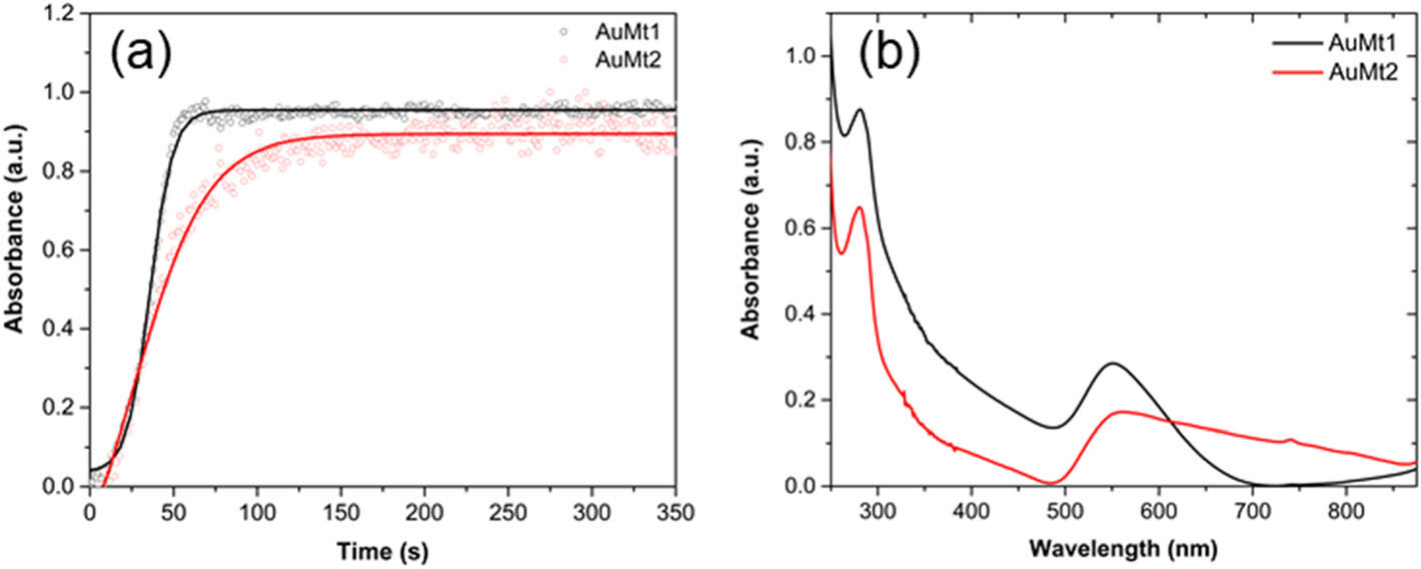
UV-Vis characterization of AuMt1 and AuMt2. **a** Kinetic formation and **b** UV-Vis spectra

Figure 2a shows a temporal evolution of absorbance on a SPR peak of AuNPs (550 and 560 nm for AuMt1 and AuMt2, respectively) as nanomaterial synthesis reaction takes place. Experimental data are fitting with Boltzmann’s sigmoidal function [52], where at least three stages of growth are observed. In the first one, absorbance grows slowly at the start of the synthesis reaction when Au^3+^ ions are reduced to Au^0^ and form aggregates of a few atoms that join to form small NPs. In the second stage, the small NPs increase their size by autocatalytic growth and absorbance grows in a fast way. In the last stage, in NP recrystallization, absorbance reaches its stationary phase. As can be seen in Fig. 2, a maximum absorbance is reached in 60 s for AuMt1 and 120 s for AuMt2. Interestingly, the first stage of growth is 20 s for AuMt1, while it is almost null (less of 1 s) for AuMt2, which is explained due to the higher proportion of reducing molecules (Mt extract) with respect to the metallic precursor. This favors the fast formation of the nucleus in NPs of AuMt2 respect to AuMt1; nevertheless, the next stage of growth of the NPs is low for AuMt2, and NPs with larger size are obtained. It has been reported that AuNPs synthesis, using maltose and tween80 as stabilizing, shows a growth kinetic with a reaction time very similar for the reported in this work [53]. In another green synthesis report [54], it is pointed out that the lowest proportion of reducing/precursor agents generates smaller size NPs.

Figure 2b shows the characteristic absorption spectra of AuMt in the region comprised of 250–875 nm. SPR for AuMt1 shows a symmetric band with a maximum absorption in 550 nm and broad of 200 nm. AuMt2 plasmon peak suffers a slight red shift localized now in 560 nm with an asymmetric band and a larger width than 300 nm, which is due to the difference in sizes between the two nanomaterials (d_AuMt1_ < d_AuMt2_)_1_  _2_. A similar behavior has been reported in AuNPs synthesis with sodium citratum as reducing agent, where the red shift and plasmon broadening are attributed to higher oscillation modes that affect the extinction cross section by increasing the NP size [55]. Additionally, both spectra show the absorption band at 280 nm corresponding to the polyphenolic molecules of the extract, which suggests that Mt extract acts as a stabilizer of the AuMtNPs.

#### Size, Zeta Potential, and Stability of AuMtNPs

AuMtNP sizes by DLS and Z-potential were tested at different conditions for one concentration (50 μg/mL) as shown in Table 1. AuMt1 and AuMt2 shown high negative values (≤ 30 mV) in water, which favor electrostatic stability of both nanoparticle systems. According to Qu et al. [56], *ζ* value gradually increases with NP size; in our case, AuMt1 has a smaller size than AuMt2 in water, a size which is controlled by NP synthesis. These size values match with NP *ζ* values, where the higher *ζ* corresponds to NP higher size. Zeta potentials (*ζ*) of AuMtNPs dispersed in s-DMEM show less negative values with respect to those obtained in ultrapure water (Table 1). This reduction can be attributed to DMEM present cations and FBS present proteins that cover AuMtNP surfaces which cause a decrease in electrostatic interactions. Despite this *ζ* reduction, the value remains close to − 25 mV for both systems which indicate that nanoparticles preserve their electrostatic stability after s-DMEM incubation [57]. Additionally, Table 1 shows the results obtained by DLS for AuMtNP hydrodynamic diameters (2R_H_) measured at 37 °C in ultrapure water and culture media. In s-DMEM, the size of both systems increased due to protein adsorption on the nanoparticle surface [58]. For AuMt1, the growth of 2R_H_ due to protein corona is 33.8 nm and for AuMt2 is 42.9 nm. It is expected that the greater the nanoparticle size will be greater than the surface for protein absorption [59]. This could explain the slightly smaller value on *ζ* for AuMt2 compared to AuMt1 in s-DMEM. For AuMt1 and AuMt2, the interaction with s-DMEM proteins is due to the extract molecules that are attached to the nanoparticle surface. These molecules differ slightly between AuMt1 and AuMt2, as shown on XPS results. We also measured the pH of solutions in the same concentration range. It was found that there is not a change in pH and whose mean value was around of 7.5 for both AuMt in ultrapure water and 7.2 in s-DMEM (Table 1).

**Table 1:** Size (PdI), zeta potential, and pH

	Size (nm)	PdI	Zeta potential (mV)	pH
AuMt1 in water (25 °C)	117.3 ± 1.07	0.195	− 35.3 ± 1.12	7.56 ± 0.16
AuMt1 in water (37 °C)	145.1 ± 8.67	0.389	–	–
AuMt1 DMEM (37 °C)	178.9 ± 7.69	0.288	− 26.5 ± 2.19	7.2 ± 0.51
AuMt2 in water (25 °C)	314.13 ± 3.00	0.172	− 42.66 ± 1.08	7.51 ± 0.35
AuMt2 in water (37 °C)	330.7 ± 11.4	0.194	–	–
AuMt2 DMEM (37 °C)	373.5 ± 9.85	0.258	− 24.8 ± 2.37	7.18 ± 0.43

Additional file 1: Figure S2 shows AuMtNP hydrodynamic diameters when dispersed in ultrapure water and s-DMEM at 37 °C, in a concentration range between 25 and 200 μg/mL. For each studied system, the hydrodynamic diameter does not change with nanoparticle concentration, and only for, AuMt2 s-DMEM at 100 μg/mL, the particle size increases with respect to the lowest evaluated concentration, which may indicate NP aggregation processes at these concentrations [32].

#### Fourier Transform Infrared Spectroscopy (FTIR)

**Fig. 3:**
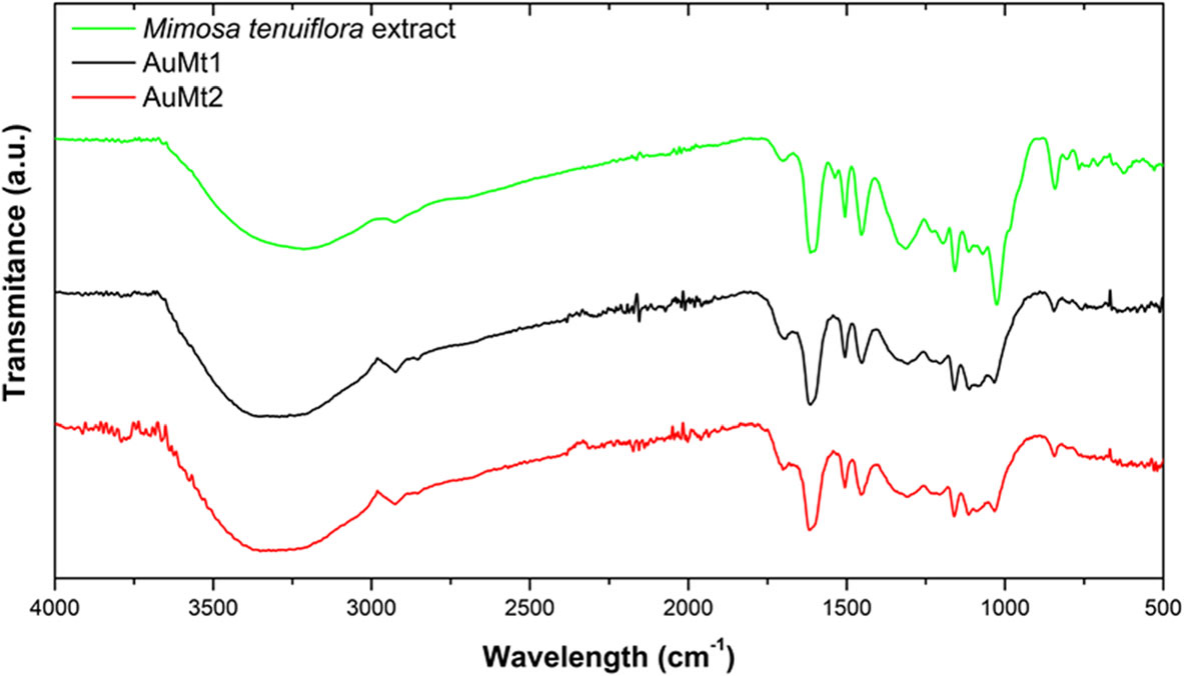
FTIR spectra. Mt extract (green), AuMt1 (black) and AuMt2 (red)

FTIR spectrum, shown in Fig. 3, corresponds to Mt extract, AuMt1, and AuMt2. The characteristic broad bands centered around 3250 cm^−1^ are associated with phenolic OH from tannins and flavonoids mainly. Peaks at 1594 cm^−1^ correspond to N-H bending vibration, at 1705 cm^−1^ to ketone acyclic stretch and region between 1000 and 1300 cm^−1^ to C–O stretch. Peaks in the range from 1600 to 500 cm^−1^ are identified with polyphenols, signals at 1235 and 1160 cm^−1^ are related with aromatic C–O bond stretching, and at 1020 cm^−1^ to aliphatic C–O band stretching and at 1235 cm^−1^ are specifically related with the characteristic of the cyclic nature of ether. These signals can be associated to the most abundant compounds in Mt extract as Mimosa tannin, flavone sakuranetin, triterpenoids saponins, chalcones, and the *N*,*N*-dimethyltryptamine alkaloid (Additional file 1: Figure S3). The samples AuMt1 and AuMt2 show the same characteristic peaks in the region of polyphenols confirming that NPs are stabilized by Mt extract molecules [60]. We observe a change of 1331 cm^−1^ in the width band and a decrease in peak intensity for the AuMt1 and AuMt2 corresponds with the bond between AuNPs and C-H group of polyphenols; 1723 cm^−1^ is shifted by oxidation of polyphenolic into carboxylic compounds during the reduction of Au^3+^ to Au^0^ [51, 61, 62].

#### X-ray Photoelectron Spectroscopy (XPS)

**Fig. 4:**
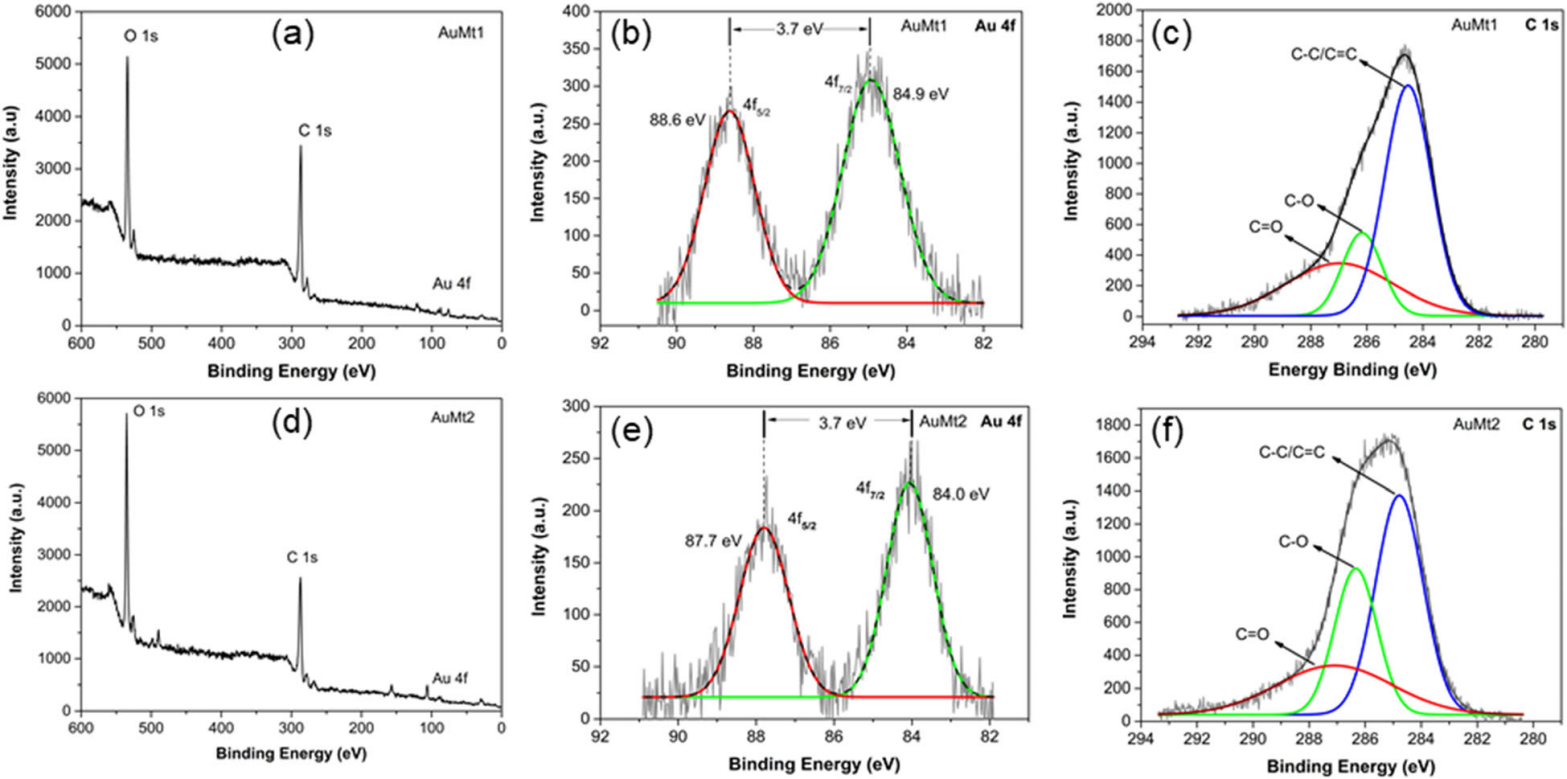
XPS spectra of AuMt1 and AuMt2. **a**, **d** Survey spectra, **b**, **e** Au4*f* high resolution, and **c**, **f** C1*s* high resolution

In the XPS survey scan analysis for AuMt1 and AuMt2, the samples have clearly shown the presence of oxygen (O 1*s*), carbon (C 1*s*) and gold (Au 4*f*), whose peaks are centered around of 532, 284, and 85 eV, respectively, as shown in Fig. 4a, b. High-resolution XPS experiments were made to establishing a relative abundance of different functional groups of molecules that coat AuNP surfaces. Au4*f* high-resolution XPS spectra for AuMt1 and AuMt2 consist of two symmetric peaks separated by 3.7 eV (Fig. 4b, e). Peaks associated with 4*f*_5/2_ spin-orbital coupling are located on binding energy (BE) of 88.6 and 87.7 eV for AuMt1 and AuMt2, respectively. For 4*f*7/2, spin-orbital coupling peaks are located on 84.9 and 84.0 eV. The link of intensities (I4*f*_7/2_ > I4*f*_5/2_) and location and separation (ΔBE = 3.7 eV) between peaks confirm that gold ions (Au^3+^) are reduced completely to metallic gold Au^0^ [63]. Au4*f* signals, for AuMt1, are slightly shifted (~ 0.9 eV) at higher energies with respect to sample AuMt2. This can be explained in terms of NP size differences between samples. AuMt1 has a half population of NPs with size less to 40 nm, while AuMt2 NPs have a mean diameter of 150 nm, determined by TEM. Peak shift for Au4*f* signals, due to the presence of small NPs, has been reported by other authors who relate the Au4*f* BE increase with decreasing NP size [64, 65]. Also, the shift effect could be due to the interaction of functional groups capped on surfaces of AuNPs [66]. In Fig. 4c, f, the high-resolution XPS spectra of C1*s* are shown for AuMt1 and AuMt2. Spectra were deconvoluted by 3 Gaussian bands associated with C=O, C–O, and C–C or C=C. For AuMt1, peaks are centered on 286.9, 286.1, and 284.5 eV, for AuMt2 on 287.0, 286.3, and 284.7 eV, respectively. Comparing the experimental XPS curves for C 1*s*, we see appreciable differences between AuMt1 and AuMt2. The main difference comes from a significant decrease in AuMt1 of the signal associated with C–O group. Comparing the percentage contributions of each group, obtained from the deconvolutions (Additional file 1: Table S2), we see that in AuMt2 contribution of C–O signal is 27.8% while in AuMt1 is 16.6%. This difference can be explained in terms of the oxide-reduction reaction that gives rise to the process of AuNP formation. The synthesis of AuMt1 is added twice the metal precursor (HAuCl_4_ is 0.01 M) than in synthesis of AuMt2. In both cases, the same amount of extract is used as a reducing agent, so in AuMt1, more hydroxyl groups (−C–OH) are consumed to reduce a greater number of Au^3+^ ions. Thus, a decrease of C–O signal in AuMt1 confirms that hydroxyl groups participate in the synthesis reaction. High-resolution XPS of O 1*s* revealed that carbonyl C=O is the most abundant group (Additional file 1: Figure S4 and Table S2). In addition, the content of the C=O group is higher in the AuMt1 sample, which confirms what was previously discussed.

XPS and FTIR indicate that AuMtNPs interact mainly with carbonyl groups (ketones) in addition to hydroxyl groups of Mimosa tannins, saponins, and other molecules that participate in the reduction of Au^3+^ to Au^0^ and stabilization of AuMtNPs [63, 67, 68].

#### Transmission Electron Microscopy

**Fig. 5:**
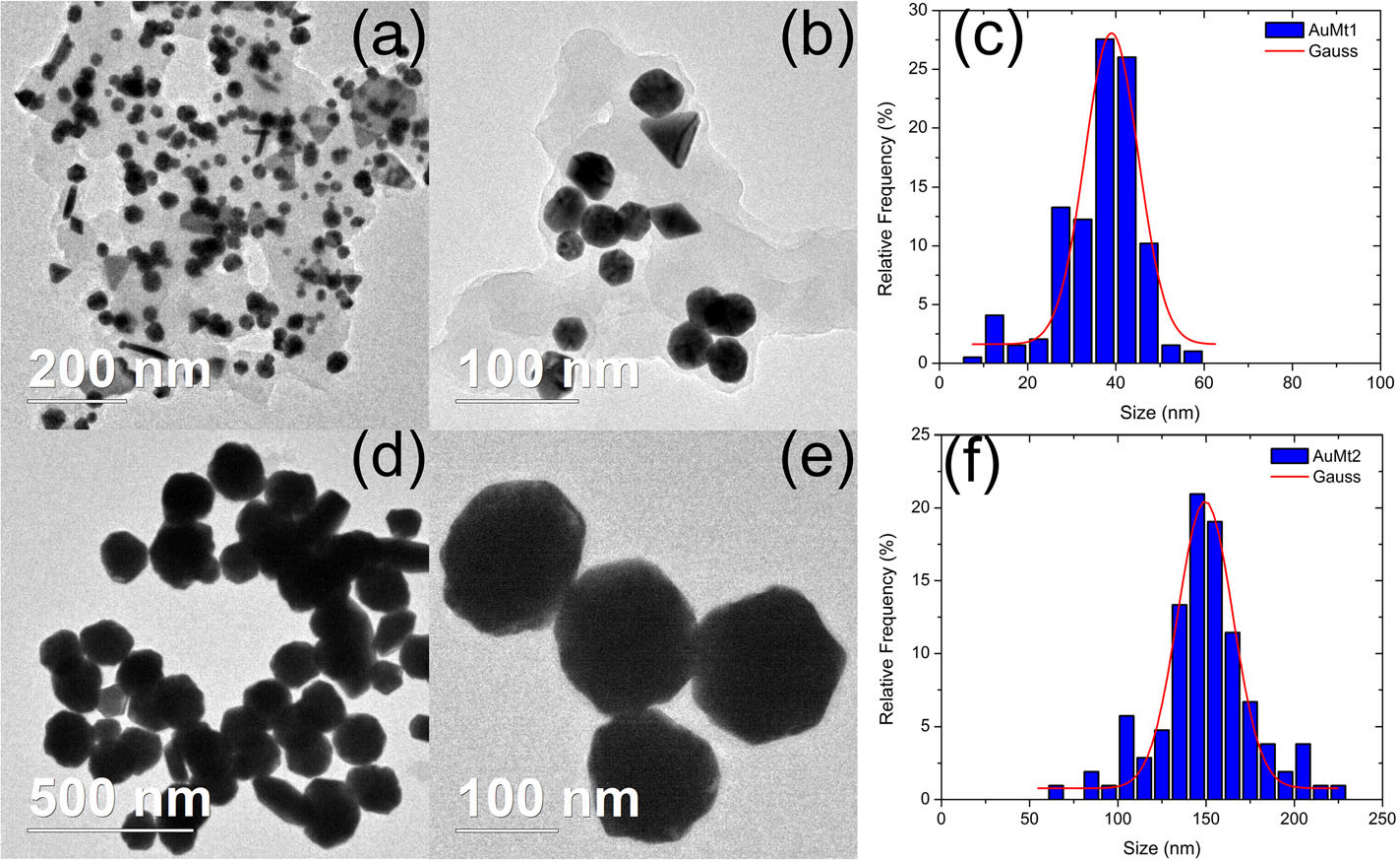
Size distributions by TEM. **a**, **b**, **c** AuMt1 and **d**, **e**, **f** AuMt2

AuMt1 TEM micrographs are shown in Fig. 5a, b and AuMt2 in Fig. 5d, e showing products’ shape distribution. AuMt1 has the biggest diversity in shapes. AuMt shape is determined by the relationship between the variation of metal precursor concentration and Mt extract at a fixed concentration. In this case, NPs were observed without cleaning the extract to observe the interaction that forms around the AuMt. As observed in the micrographs, an extract is placed on the surface; however, NPs are kept dispersed and no aggregation is shown. Figure 5c, f show size distribution for each sample, and AuMt1 have an average size dispersion of 40 nm and AuMt2 of 150 nm.

**Fig. 6:**
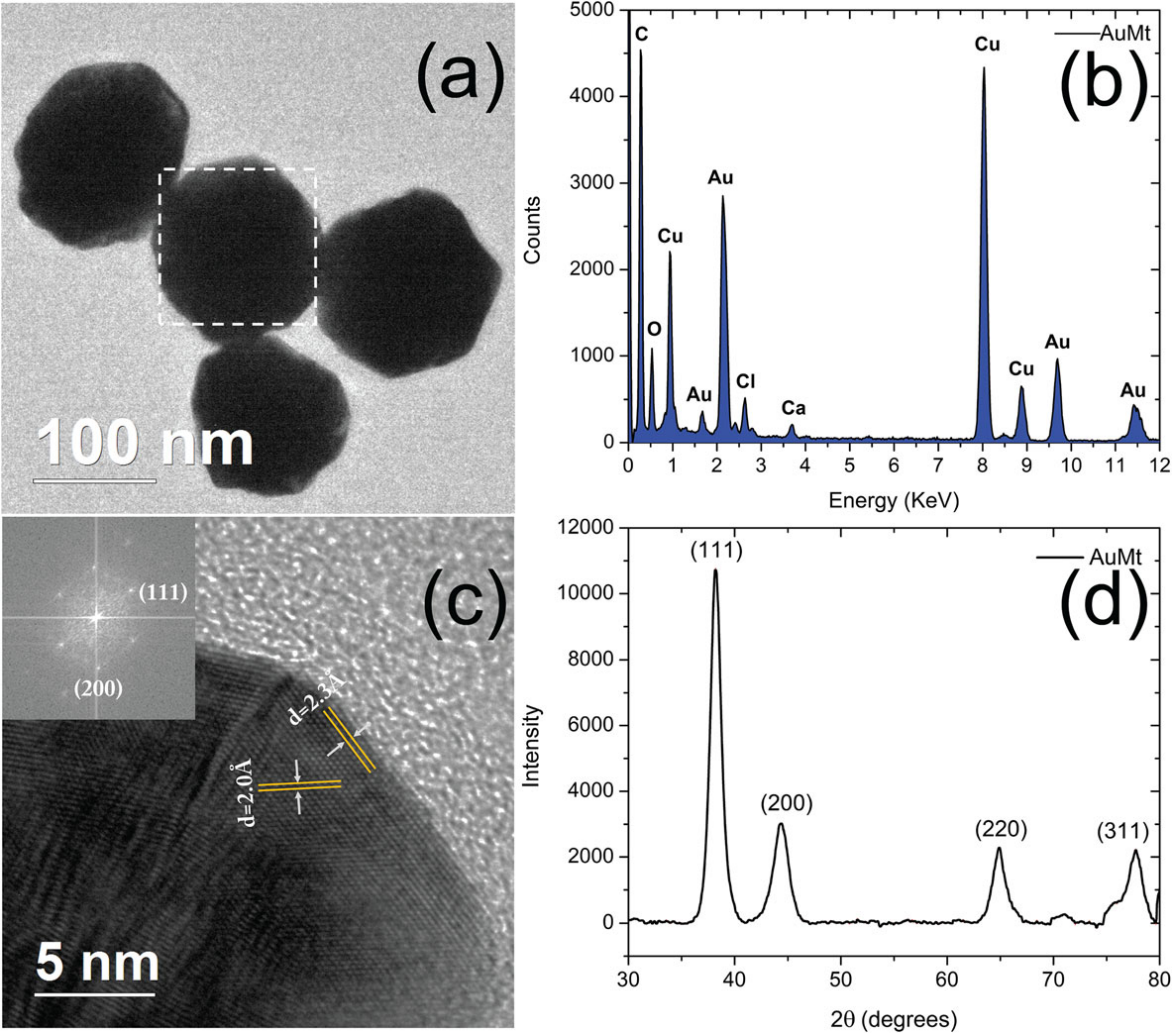
Nanostructural characterization of AuMt. **a** TEM, **b** EDS, **c** single HRTEM, and FFT and **d** XRD

In Fig. 6a, AuMt TEM micrographs were also analyzed by EDS (Fig. 6b), which showed Au presence. Other chemical elements such as Cl, O, and Ca, on EDS spectrum, come from the extract that surrounds NPs. According to the crystallographic tab (JCPDS file: 04-0784), the obtained distances between 2.35 and 2.03 Å (Fig. 6c) correspond to Au crystalline planes (111) and (200).

#### X-ray Diffraction (XRD)

Figure 6d shows the characteristic AuMt XRD diffraction peak at 2*Ɵ*, which are in 38.17, 44.37, 64.81, and 77.66^o^ corresponding with the planes (111), (200), (220), and (311), respectively; these planes correspond with the face-centered cubic Au (space group Fm^3^m, JCPDS File No. 89-3722). High Score Plus and Origin software were used for the analysis [69].

### Biological Tests

#### Cytotoxicity by MTT and Live/Dead Assay

**Fig. 7:**
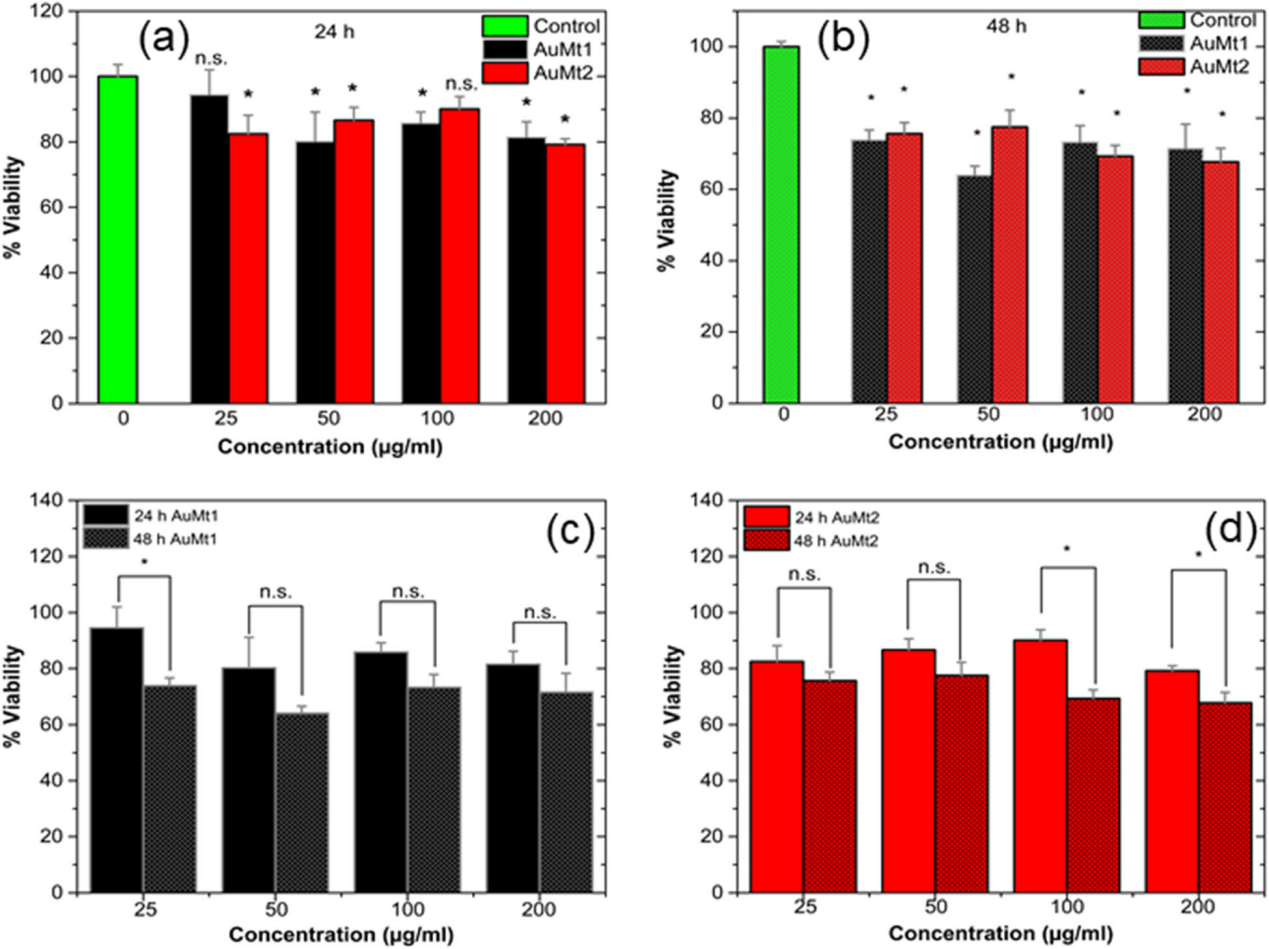
Viability assay using MTT in HUVEC cell. **a** For 24 h and **b** 48 h. 0ne-way ANOVA analysis with (*p < 0.05). Comparison between 24 and 48 h for **c** AuMt1 and **d** AuMt2 with ANOVA analysis with Tukey tests (n.s. no significance and (*p < 0.05))

To evaluate AuMt1 and AuMt2 toxicity, tests were performed on HUVEC cells using MTT. Four concentrations and two times for both materials were evaluated. In Fig. 7a, it is observed that at 24 h for AuMt1, cell viability decreases between 10 and 20%, only in concentrations higher than 25 μg/mL. For AuMt2, a similar effect is obtained in cell viability; however, the concentration of 100 μg/mL seems to have no effect on these tests. In Fig. 7b, MTT tests at 48 h for AuMt1 and AuMt2 are shown. For AuMt1, it is easy to notice that concentration with the greatest effect is 50 μg/mL, where the viability drops almost 30% compared to the control. The concentration of 50 μg/mL seems to be the concentration with the highest toxic effect; however, when the obtained data were analyzed, it is found that there is no significant difference between the obtained data on 24 and 48 h, a similar result obtained for 100 and 200 μg/mL, Fig. 7c. For AuMt2, a toxic effect between 20 and 30% is observed only on 100 and 200 μg/mL, while 25 and 50 μg/mL show no significant difference, compared to the observed effect at 24 h, Fig. 7d. This seems to correlate with AuMt2 size growth in s-DMEM (Additional file 1: Figure S2) where at a concentration of 100 μg/mL, they begin to aggregate. In the work published by Chandran et al. [70], they used gold nanoparticles coated with branched polyethyleneimine (BPEI), lipoic acid (LA), and polyethylene glycol (PEG), where they see an important toxicity in HUVEC cells by nanoparticles coated with BPEI, which have sizes of 40 and 80 nm, where viability is between 20 and 30%. When these particles are covered with human serum proteins, it is found that toxicity decreases; this is due to the corona effect. Recently, Zhaleh et al. [71] have reported the biogenic synthesis of 40-nm gold nanoparticles using leaf extracts from *Gundelia tournefortii L.* plant. Interestingly and in contrast to our results, the authors indicate that MTT cell viability tests for these particles in HUVEC, the cell viability was 95% at 1000 μg/mL; however, they do not establish if the low cytotoxicity is due to the fact that there is no material internalization or if the particles are harmless due to protein corona. In this sense, bioreductive compounds present in *Gundelia tournefortii L* extract are different from those reported for *Mimosa tenuiflora* extract (Additional file 1: Figure S3). Thus, the interactions of these two nanoparticle systems with proteins present in FBS are very different, which may explain the differences in cytotoxic responses.

As mentioned above, AuMt1at a 50-μg/mL concentration shows the highest toxicity and cellular uptake. We believe that toxicity may be due to the fact that the nanomaterial has a low affinity to s-DMEM proteins, since it has only 16.6% of hydroxyl groups on the surface to promote hydrogen bonding with S-DMEM proteins. The fact that the material toxicity decreases as AuMt1 concentration increases may be due to a cellular detoxification response, like an exocytosis caused by high intracellular content of gold [70]. For AuMt2, the toxicity effect at 100 and 200 μg/mL may be due to nanomaterial agglomeration, which could be attaching to the membrane causing adverse effects for the cells; however, more experiments are required to confirm this hypothesis.

**Fig. 8:**
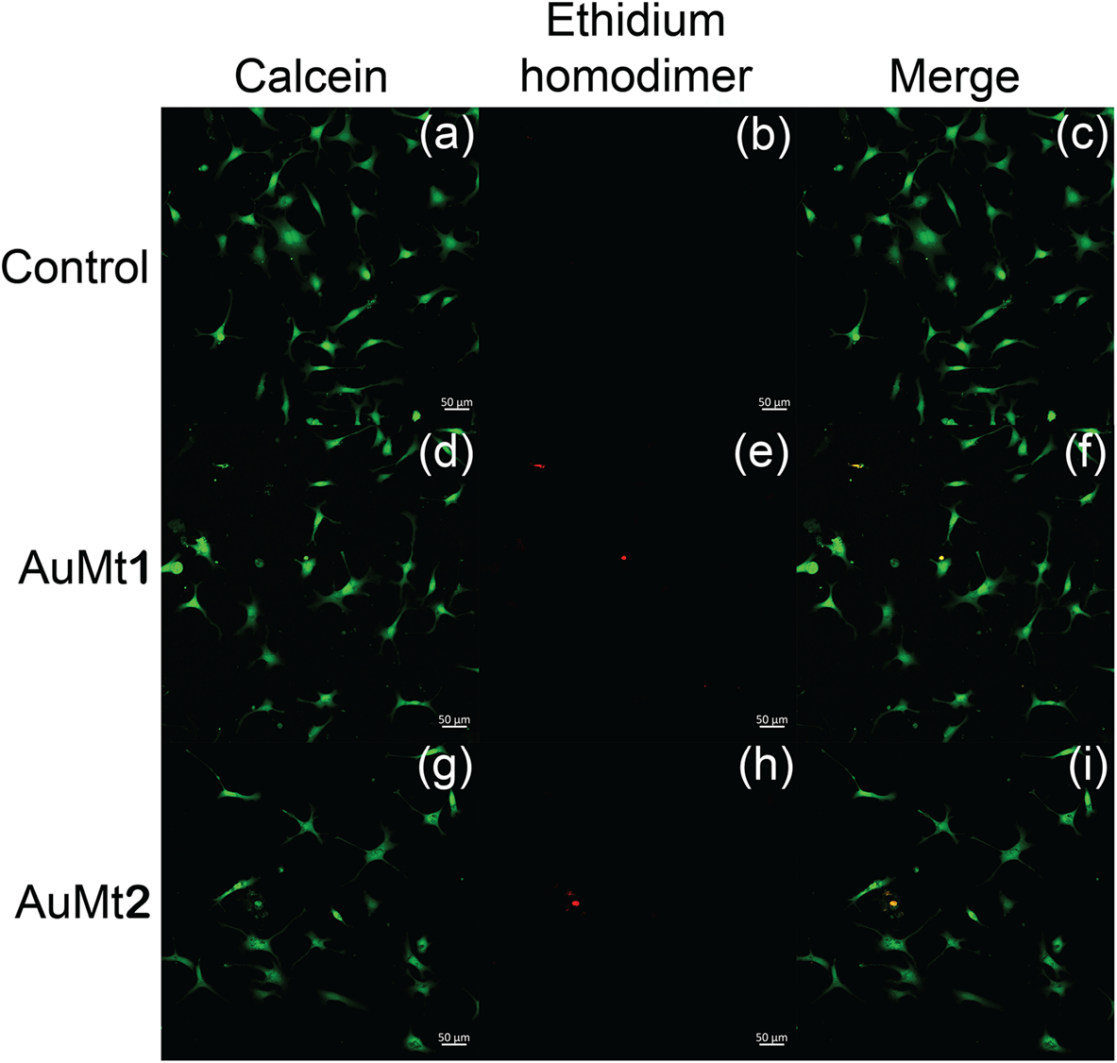
Live/dead assay in HUVEC cells. **a**, **d**, and **g** with calcein; **b**, **e**, and **h** with ethidium homodimer; and **c**, **f**, and **i** merge by confocal microscopy

Only one concentration (50 μg/mL) was chosen to be evaluated by live/dead fluorescent dye; this is due to the purpose of confirming the MTT results and later analyzing the metallic NP internalization in HUVEC cells, avoiding a field saturation by NPs. When cells were stained with live/dead fluorescent dye kit, it was found that a large part of the cell population favorably marked for calcein and just a few for ethidium homodimer, indicating that cell culture is viable, as shown in Fig. 8.

#### Confocal Laser Scanning Microscopy: Fluorescence of AuMt

**Fig. 9:**
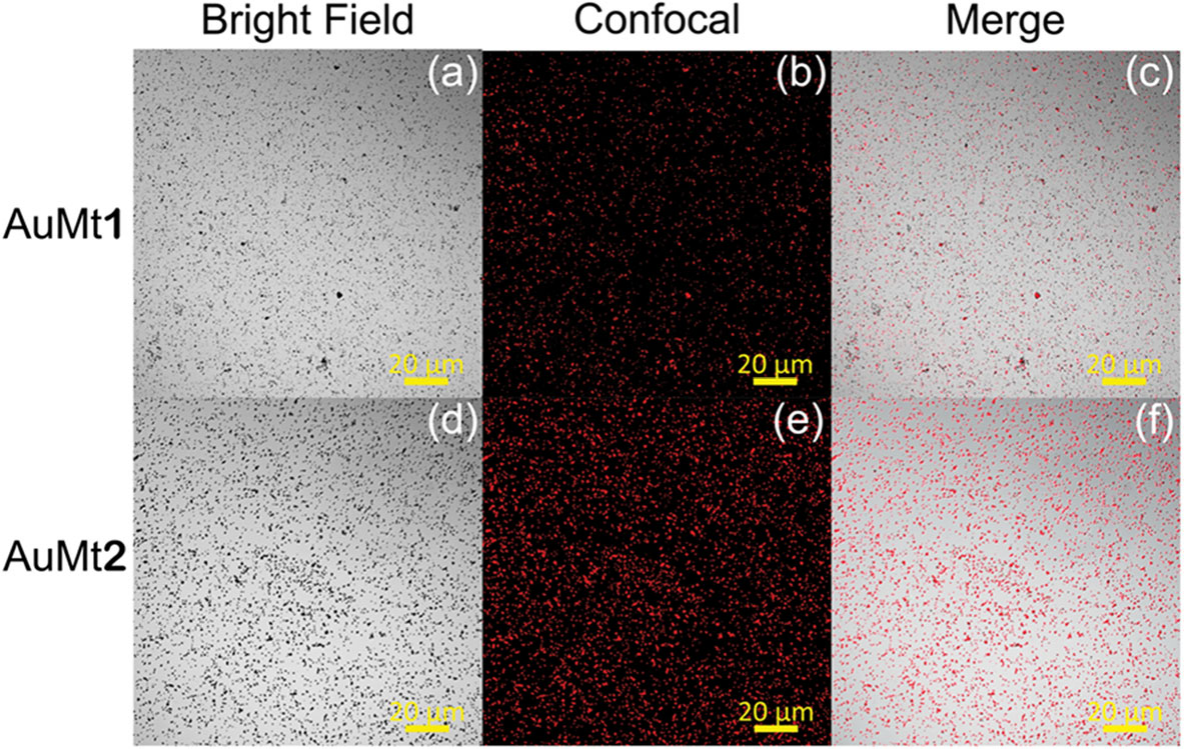
AuMt1 and AuMt2 fluorescence. **a**, **d** Bright field. **b**, **e** Confocal. **c**, **f** Merge

In Fig. 9a, d are shown micrographs of AuMt1 and AuMt2 in bright field and in Fig. 9b, e, their corresponding fluorescence, captured by confocal microscopy. Red fluorescence of AuMt (collected emission 650–700 nm) was excited employing 640 nm diode laser, and a mayor size of NPs can be appreciated in AuMt2 sample than AuMt1. In the merge images in Fig. 9c, f, it can be observed how the luminescence comes exclusively from the dark points associated with the NPs. This indicates that the cleaning process effectively removed the extract that is not complexed to the nanomaterial, so there is no background emission. It is interesting to observe that an intense fluorescence of the NPs captured by the confocal system is achieved at a very low excitation power of the laser (below 0.5 mW). So, this NPs system can be fluorescently traced efficiently in cellular systems with little risk of phototoxicity. Some authors have reported fluorescent emission about 610 nm, suggesting intrinsic Au fluorescence [72, 73]. AuNPs emission is related to the core size confinement effect that generates discreet electronic states [74]. However, in our case, the metal surface is covered with flavonoids, which show fluorescence, and when complexing with AuNPs, the fluorescence of both is enhanced. Different authors have reported that fluorescence is largely enhanced by charge transfer from the surface ligands to the metal core via S–Au bonds [75]. It has also been reported that ligands (thiol molecules, DNA oligonucleotides, dendrimers, polymers, peptides, and proteins) affect AuNPs optical and electronic properties since its fluorescent properties can be significantly affected by their surface chemistry [76].

#### Cellular Internalization

**Fig. 10:**
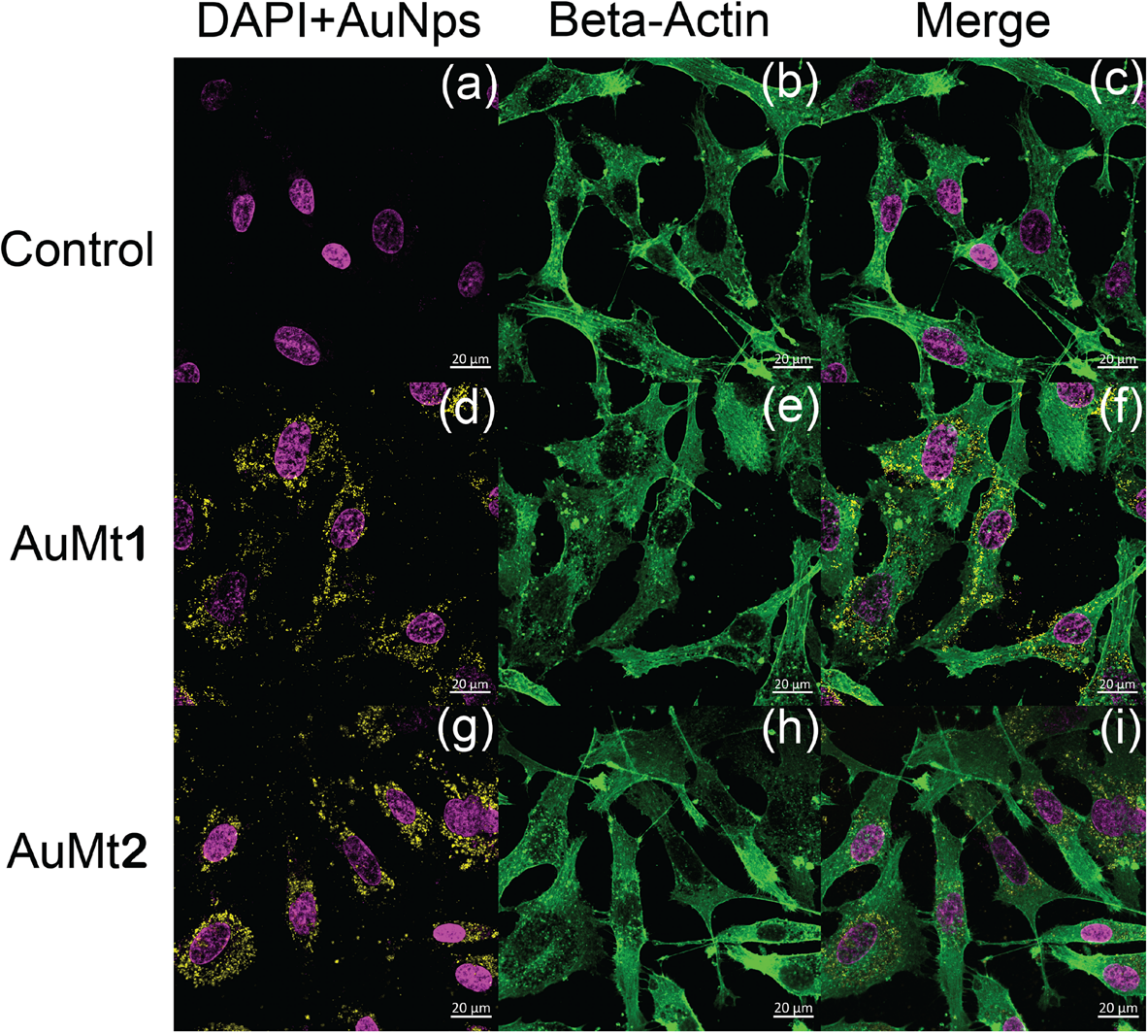
AuMt1 and AuMt2 cellular internalization. **a**, **d**, and **g** with DAPI; **b**, **e**, and **h** with beta-actin; and **c**, **f**, and **i** merge by confocal microscopy

Cells were also analyzed, by confocal microscopy, after 24 h incubation, with AuMt1 and AuMt2 at a concentration of 50 μg/mL. The nucleus is shown in blue color using DAPI Fig. 10a, and the cytoskeleton structure was stained with anti-beta actin in green color Fig. 10b and merge Fig. 10c. The observed micrographs were obtained through 3 different channels on separate tracks, where the excitation wavelengths were 405, 488, and 640 nm for DAPI, anti-beta actin, and AuMt, respectively.

**Fig. 11:**
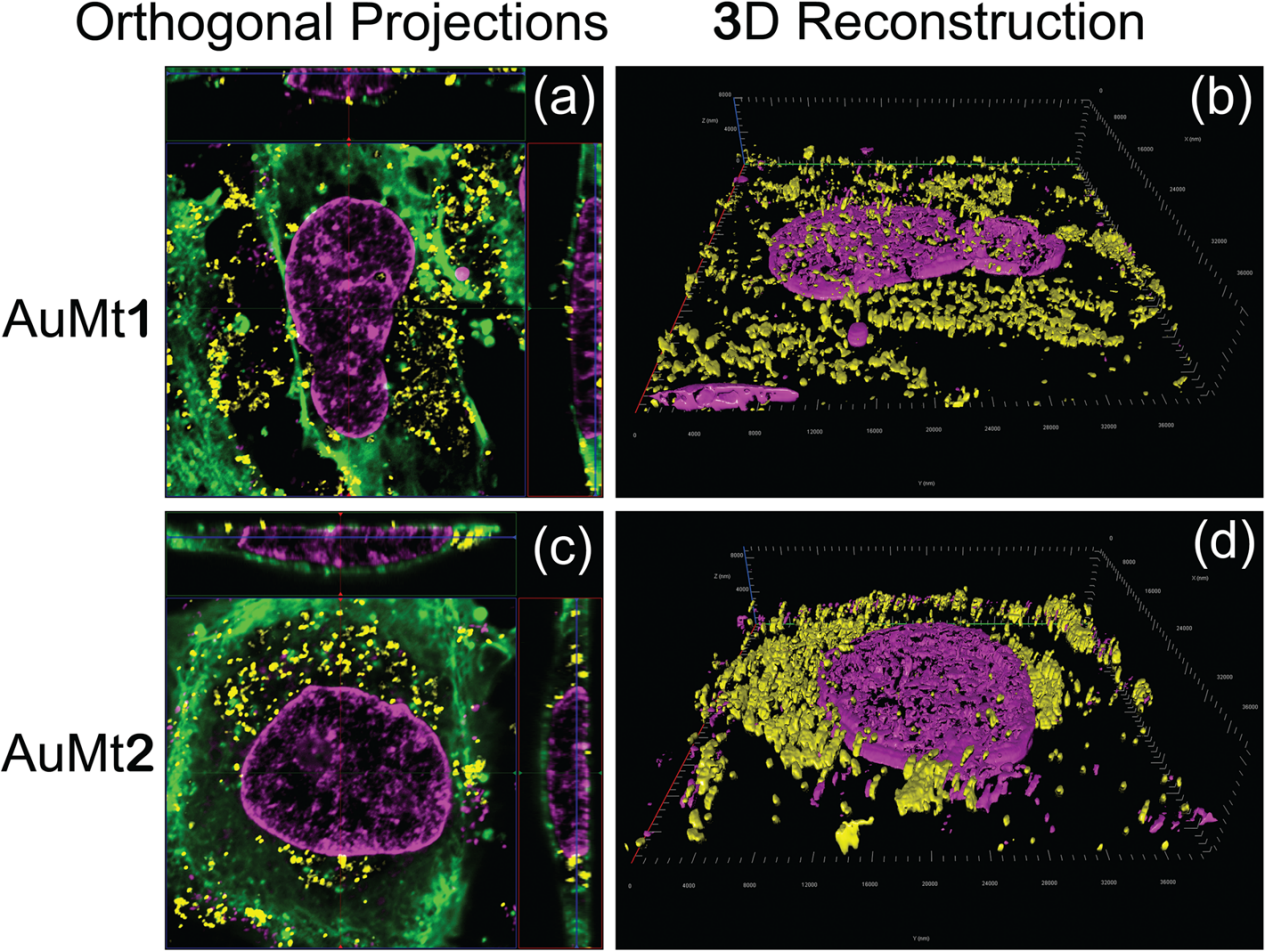
Orthogonal projections and 3-D images. **a**, **c** AuMt1 and AuMt2 cellular internalization analysis through orthogonal projection and **b**, **d** 3-D reconstruction by confocal microscopy

As previously described, cells were also analyzed by confocal microscopy, for AuMt1 and AuMt2 internalization, at a concentration of 50 μg/mL. Confocal micrographs show that AuMt are internalized in HUVEC cells cytosolic space, and many of these particles are surrounding the nucleus, without being internalized in it. When not observing particles in the nucleus, a more meticulous analysis was carried out, by cell orthogonal projection and a 3-D reconstruction. Observing the micrographs of both reconstructions, it is possible to notice that AuMt is distributed differentially. In Fig. 11a, b for AuMt1, it can be observed that a material is dispersed in the cytoplasm, while in AuMt2, the material is concentrated in the nuclear periphery, as shown in Fig. 11c, d. We were not able to find NPs in the nucleus, and this suggests that the nanomaterial has little or no genotoxic potential, since it has no way of interacting with nuclear DNA, which is a quality for a nanocarrier. Efficient cellular uptake depends on NP size, shape, charge, and coating, the parameters that can affect their interactions with cell proteins. The fact that polyphenolic compounds are found on AuMt surface could facilitate the nanomaterial internalization, which would make it a candidate as a possible pharmacological nanocarrier [77–79].

The obtained results for an AuMtNP cellular uptake in HUVEC by a confocal microscopy at 50 μg/mL suggests that AuMt1 interacts with the cells in a greater quantity than AuMt2 in a 3:1 ratio, as seen in Additional file 1: Figures S5–S7. If we consider that protein corona in AuMt1 is 9.1 nm smaller than in AuMt2, we can suggest that AuMt1-efficient internalization by HUVEC cells is given by a combination of factors such as AuMt1 smaller size, the highest absolute value of *z* potential and the lower thickness of protein corona. This indicates a poor protein coverage that allows partial exposure of the nanoparticle surface, which is rich in extract molecules. Therefore, nanoparticles can interact by means of extract molecules with surface-specific membrane receptors that facilitate the internalization of AuMt1.

### Catalytic Tests

#### Catalysis

Analysis of catalytic reaction was realized to calculate the degradation percentage *(*%*D)* using the Eq. (5):

5 $$ \%D=\frac{\left({A}_0-A\right)}{A_0}x100 $$

using the *A*_0_ absorbance at *t* = 0 and *A* is the absorbance at time *t*. Langmuir-Hinshelwood equation was used to calculate the slope of the regression plot $\ln \left(\frac{A}{A_0}\right)$ versus irradiation time [80], which is expressed in Eq. (6) and *K* is the first-order rate constant of the degradation ratio:

6 $$ \ln \left(\frac{A}{A_0}\right)= Kt $$

**Fig. 12:**
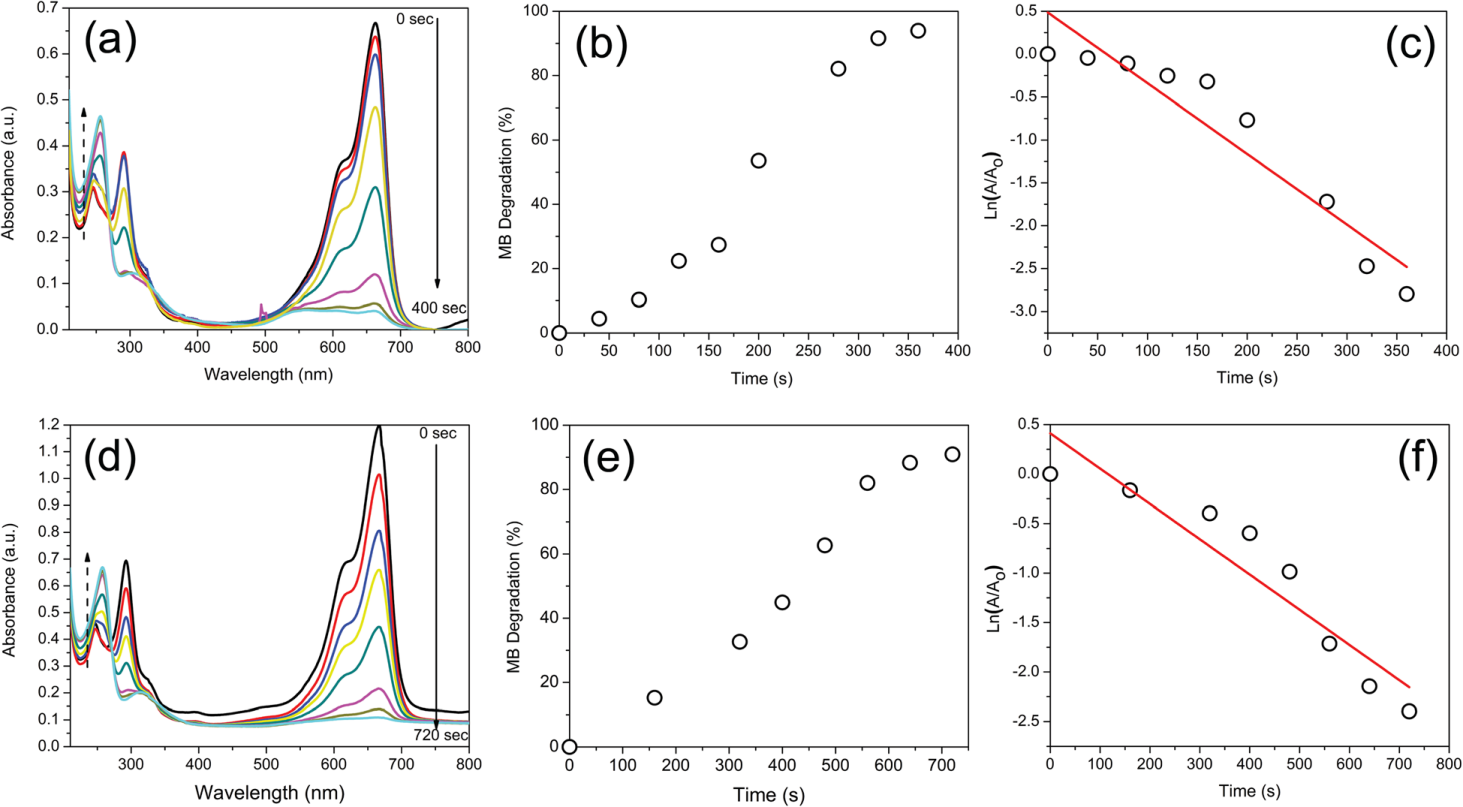
AuMt1 and AuMt2 Catalysis. **a**, **d** UV-Vis spectra. **b**, **e** Percentage. **c**, **f** Ratio of MB degradation

For the analysis of catalytic activity on MB degradation, the absorbance at 660 nm was monitored. Figure 12 shows the AuMt1 and AuMt2 catalytic activity, where a decrease on maximum absorption of MB is observed as time progresses Fig. 12a, d. MB degradation and its conversion to leucomethylene is confirmed by progressive decreases of the absorbance at 292, 614, and 660 nm correspond to MB and by the increase in time of the absorbance at 256 nm associated with leucomethylene. Homogeneous catalysis reaches a 50% MB degradation at 190 s Fig. 12b, while the degradation ratio *K* for the total process is 8.24 × 10^−3^s to AuMt1 Fig. 11c. AuMt2 reaches a 50% of MB degradation in 400 s, Fig. 12e, and *K* takes the value of 3.54 × 10^−3^/s, Fig. 12f.

On this way, AuMt1 have a more efficient response than AuMt2. We observed a size-dependent effect (AuMt) in degradation ratio [81], and a total surface area of NPs is inversely proportional to the NP size [37]. Table 2 shows a comparison between different green syntheses of AuNPs and their *K* obtained in size function.

**Table 2:** Comparison of *K* for degradation of MB using AuNPs as a size function

Reducing and stabilizing agent	Sizes (nm)	Rate constant *K* (1/min)	Reference
Kashayan	15 to 50	0.66801	[82]
		0.33055	
		0.1318	
Pogestemon benghalensis	10 to 50	0.1758	[29]
Salmalia malabárica gum	12	0.241	[83]
Mimosa tenuiflora	40 and 150	0.4944	
		0.2124	

## Conclusions

In this work, we show for the first time that the extracts of bark of Mimosa tenuiflora allow the production at room temperature of gold nanoparticles by means of one-pot synthesis. AuNP sizes are easily controlled by regulating a metal precursor/reducing an extract ratio. It was observed that AuMt1 and AuMt2 cellular uptakes generate a moderate cytotoxic effect at 24 and 48 h post exposition. However, toxicity does not behave in a dose-dependent manner, which suggests different action mechanisms for AuMt1 and AuMt2. XPS and FTIR indicate that AuMtNPs interact mainly with carbonyl groups (ketones) in addition to hydroxyl groups of Mimosa tannins, saponins, and other molecules that participate in the reduction of Au^3+^ to Au^0^ and stabilization of nanomaterials. Polyphenols adsorbed on AuMtNPs facilitate nanoparticle internalization. AuMt2 were located near the nuclear periphery, but for AuMt1, it was observed that nanoparticles distribute on the whole cell and present a 3 fold uptake in comparison to AuMt2. Due to the fluorescence property at low excitation power and a high cellular uptake, AuMtNPs synthesized with Mt bark extracts are candidates for its implementation as drug nanocarriers and fluorescent probes in cells. However, other strategies must be addressed, in order to reduce the nanomaterial toxicity. Finally, it was observed that AuMtNPs showed a relevant catalytic activity on MB degradation using NaBH4 as a reducing agent.

## Supplementary information

**Additional file 1: Table S1.** Formulations used in AuMt1 and AuMt2 synthesis. **Figure S1.** Time-dependent pH change of AuMtNPs synthesis. The inset shows mechanism for the reduction of gold ions into Au^0^ in presence of polyphenolics groups. **Figure S2.** DLS of AuMt1 and AuMt2 in water and sDMEM at 37 ^o^C. **Figure S3.** Main reported compounds of *Mimosa tenuiflora*. **Figure S4.** Deconvolution signal of XPS O 1s of AuMt1 and AuMt2. **Table S2.** XPS Peak fitting gaussian parameters. **Figure S5.** Region of Interest (ROI) of AuMt1 in HUVEC for Uptake NPs in cells. **Figure S6.** ROI of AuMt2 in HUVEC for Uptake NPs in cells. **Figure S7.** Fluorescence Intensity for AuMt1 and AuMt2 in HUVEC cells obtained for confocal microscopy.

## Data Availability

All datasets are presented in the main paper.

## Abbreviations

ANOVA: Analysis of variance
AuMt: Colloids formed by AuNPs and molecules of Mt
AuNPs: Gold nanoparticles
CLSM: Confocal laser scanning microscopy
DLS: Dynamic light scattering
DMEM: Dulbecco’s modified Eagle medium
DMSO: Dimethyl sulfoxide
DPPH: 2,2-Diphenyl-1-picrylhydrazyl
EDS: Energy dispersive X-rays spectroscopy
FTIR: Fourier transform infrared spectroscopy
HAuCl_4_: Tetracloroauric acid
HRTEM: High-resolution TEM
HUVEC: Human umbilical vein endothelial cells
MB: Methylene blue
Mt: *Mimosa tenuiflora*
MTT: 3-(4,5-Dimethylthiazolyl-2)-2,5-diphenyltetrazolium bromide
NaBH_4_: Sodium borohydride
PBS: Phosphate-buffered saline
PdI: Polydispersity index
PMT: Photomultiplier tube
ROI: Region of interest
SD: Standard deviations
SPR: Surface plasmon resonance
TEM: Transmission electron microscopy
UV-Vis: Ultraviolet-visible
XPS: X-ray photoelectron spectroscopy
XRD: X-ray diffraction
