# Supplementary material for: Synthesis of Gold Nanoparticles Using Mimosa tenuiflora Extract, Assessments of Cytotoxicity, Cellular Uptake, and Catalysis
Source: Nanoscale Res Lett. 2019 Oct 26;14:334. doi: 10.1186/s11671-019-3158-9 (PMC6814701; doi:10.1186/s11671-019-3158-9)
Supplement: Supplementary file 1 — Additional file 1: Table S1. Formulations used in AuMt1 and AuMt2 synthesis. Figure S1. Time-dependent pH change of AuMtNPs synthesis. The inset shows mechanism for the reduction of gold ions into Au0 in presence of polyphenolics groups. Figure S2. DLS of AuMt1 and AuMt2 in water and sDMEM at 37 oC. Figure S3. Main reported compounds of Mimosa tenuiflora. Figure S4. Deconvolution signal of XPS O 1s of AuMt1 and AuMt2. Table S2. XPS Peak fitting gaussian parameters. Figure S5. Region of Interest (ROI) of AuMt1 in HUVEC for Uptake NPs in cells. Figure S6. ROI of AuMt2 in HUVEC for Uptake NPs in cells. Figure S7. Fluorescence Intensity for AuMt1 and AuMt2 in HUVEC cells obtained for confocal microscopy. [file 11671_2019_3158_MOESM1_ESM.docx]

Additional file 1

**Synthesis of Gold Nanoparticles Using Mimosa tenuiflora Extract, Assessments of Cytotoxicity, Cellular Uptake and Catalysis**

Ericka Rodríguez-León^1^, Blanca E. Rodríguez-Vázquez^2^, Aarón Martínez-Higuera^1^, César Rodríguez-Beas^1^, Eduardo Larios-Rodríguez^3^, Rosa E. Navarro^2^, Ricardo López-Esparza^1^ and Ramón A. Iñiguez-Palomares^1*^

^1^ Physics Department, University of Sonora, Rosales and Transversal 83000, Hermosillo,

Sonora, Mexico.

^2^ Polymer and Material Department, University of Sonora, Rosales and Transversal 83000,

Hermosillo, Sonora, Mexico.

^3^ Chemical Engineering and Metallurgy Department, University of Sonora, Rosales and

Transversal 83000, Hermosillo, Sonora, Mexico.

** Corresponding author: E-mail:* [*ramonalfonso.iniguez@gmail.com*](mailto:ramonalfonso.iniguez@gmail.com)

**Table S1** Formulations used in AuMt1 and AuMt2 synthesis

| **Sample** | **HAuCl_4_** 0.01M  V (mL)  pH=2.02 | **Mt Extract**  121.8 mg/mL  Vol (mL)  pH=5.10 | **Ultrapure Water**  Vol (mL)  pH=6.47 |
| --- | --- | --- | --- |
|  |  |  |  |
| AuMt1 | 3,2 | 1,6 | 1,20 |
|  |  |  |  |
| AuMt2 | 1,6 | 1,6 | 2,8 |

*
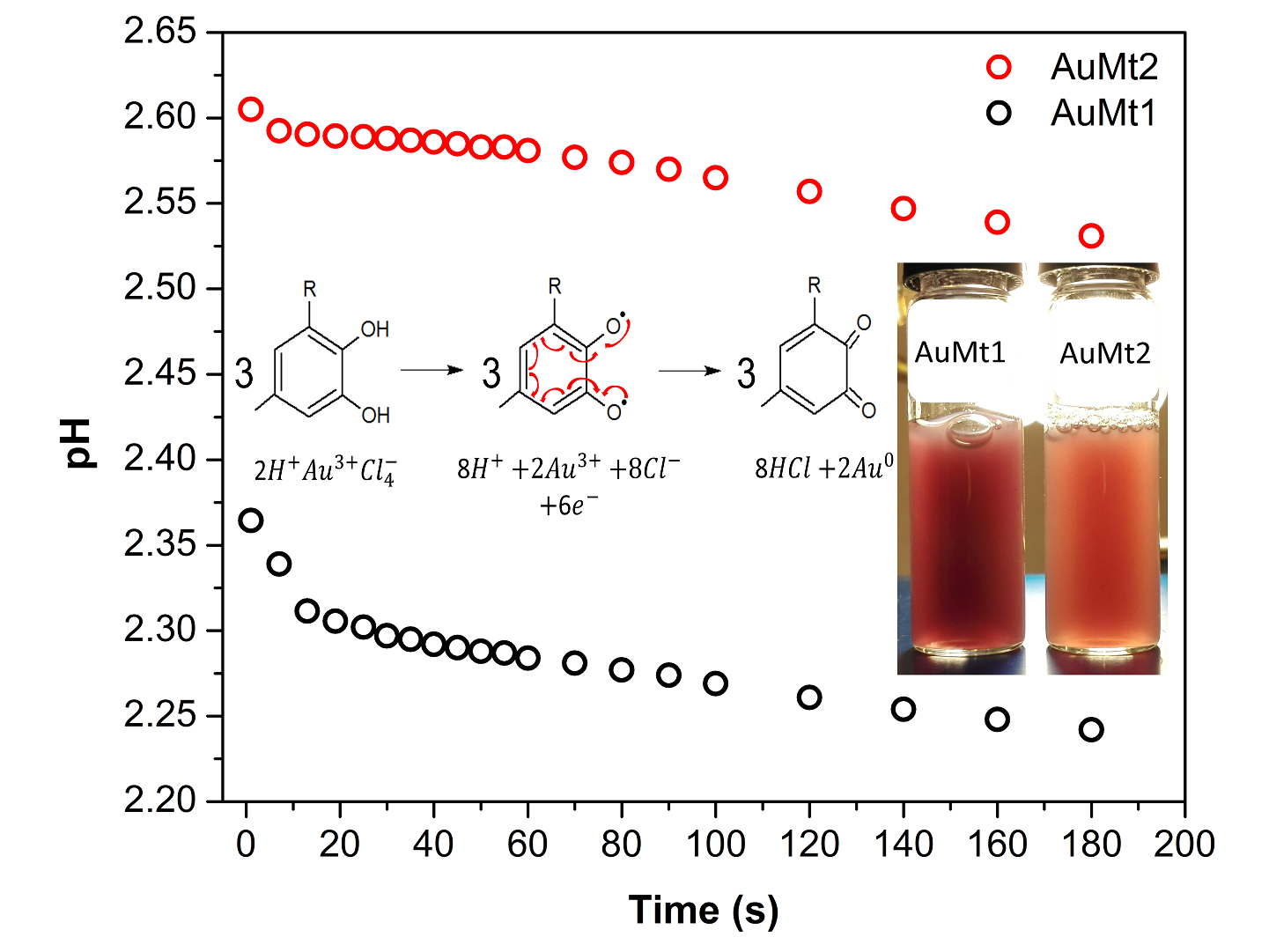
*

**Figure S1**. Time-dependent pH change of AuMtNPs synthesis. The inset shows mechanism for the reduction of gold ions into Au^0^ in presence of polyphenolics groups

**

**

**Figure S2.** DLS of AuMt1 and AuMt2 in water and sDMEM at 37 ^o^C

*
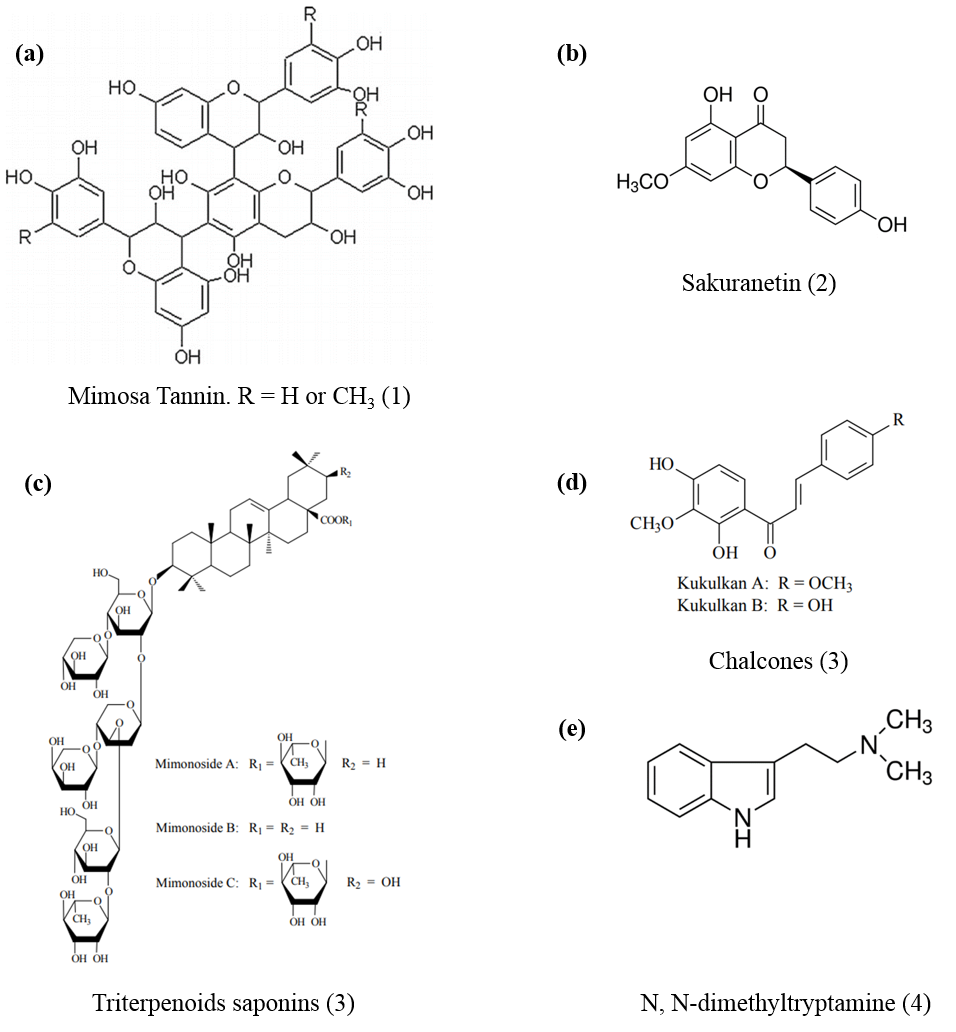
*

**Figure S3.** Main reported compounds of *Mimosa tenuiflora*.


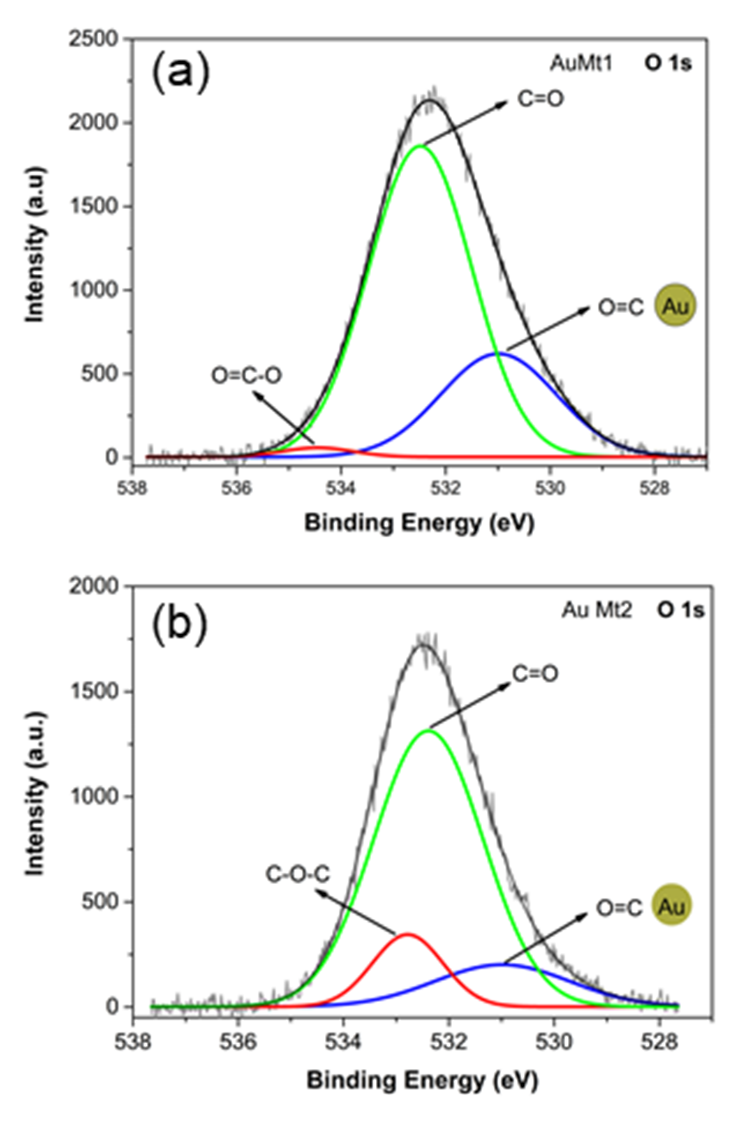


**Figure S4.** Deconvolution signal of XPS O 1s of AuMt1 and AuMt2.

**Table S2.** XPS Peak fitting gaussian parameters

|  |  | **AuMt1** | | | **AuMt2** | | |
| --- | --- | --- | --- | --- | --- | --- | --- |
|  |  | Xc (eV) | FMWH | A (%) | Xc (eV) | FMWH | A (%) |
| **Au 4f** | 4f_5/2_ | 88.63 | 1.46 | 46.26 | 87.79 | 1.48 | 45.81 |
|  | 4f_7/2_ | 84.95 | 1.51 | 53.74 | 84.05 | 1.38 | 54.19 |
| **C 1s** | C=O | 286.97 | 4.51 | 28.61 | 287.09 | 1.95 | 25.45 |
|  | C-O | 286.17 | 1.65 | 16.63 | 286.33 | 1.65 | 27.84 |
|  | C-C/C=C | 284.53 | 1.95 | 54.74 | 284.78 | 4.51 | 46.68 |
| **O 1s** | O=C-O | 534.44 | 1.61 | 1.47 | ----- | ----- | ----- |
|  | C-O-C | ----- | ----- | ----- | 532.78 | 1.57 | 13.86 |
|  | C=O | 532.48 | 2.32 | 71.85 | 532.38 | 2.45 | 12.4 |
|  | O=C | 530.98 | 2.6 | 26.67 | 530.98 | 3.02 | 73.74 |

where Xc is the Gaussian center, A is the area under curve and FMWH is Full Width at Half Maximum.

**
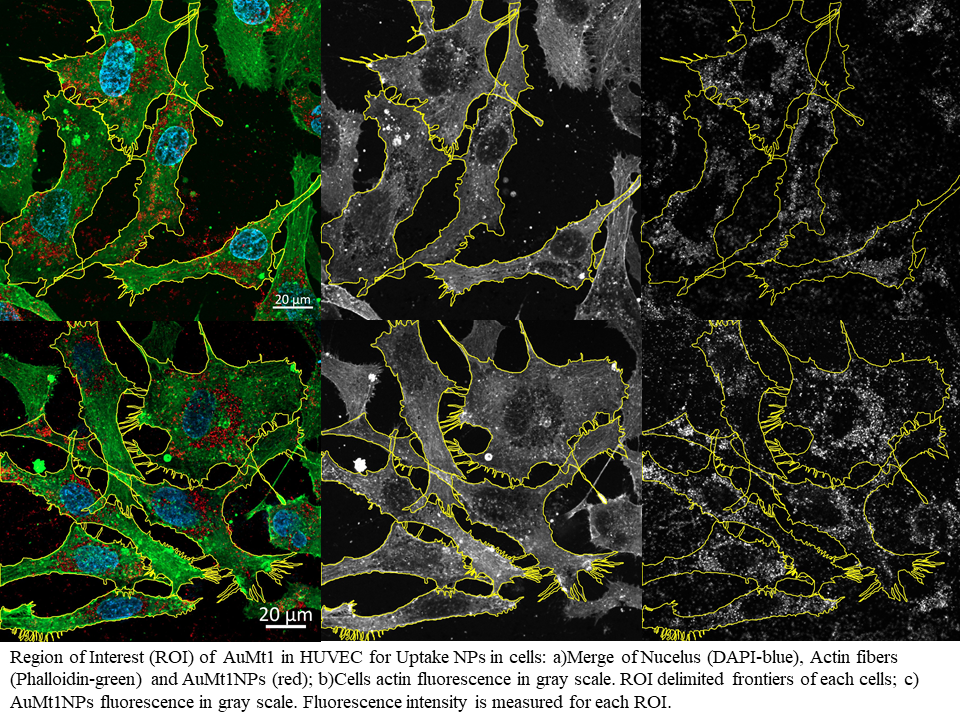
**

**Figure S5.** Region of Interest (ROI) of AuMt1 in HUVEC for Uptake NPs in cells: a)Merge of Nucleus (DAPI-blue), Actin fibers (Phalloidin-green) and AuMt1NPs (red); b)Cells actin fluorescence in gray scale. ROI delimited frontiers of each cells; c) AuMt1NPs fluorescence in gray scale. Fluorescence intensity is measured for each ROI.

**
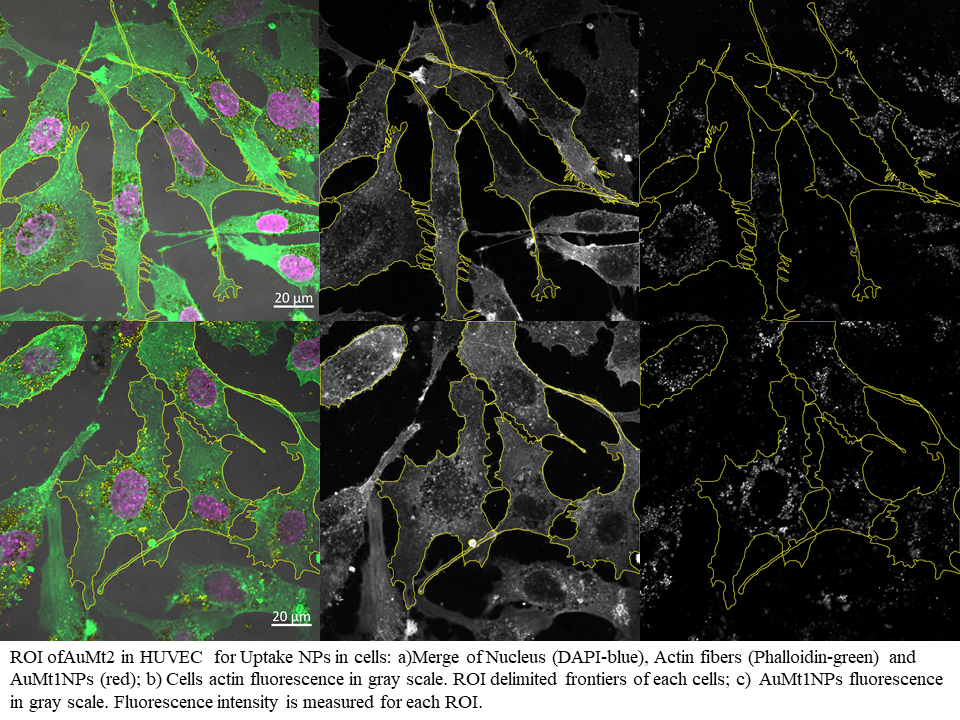
**

**Figure S6.** ROI of AuMt2 in HUVEC for Uptake NPs in cells: a) Merge of Nucleus (DAPI-blue), Actin fibers (Phalloidin-green) and AuMt1NPs (red); b) Cells actin fluorescence in gray scale. ROI delimited frontiers of each cells; c) AuMt1NPs fluorescence in gray scale. Fluorescence intensity is measured for each ROI.

**
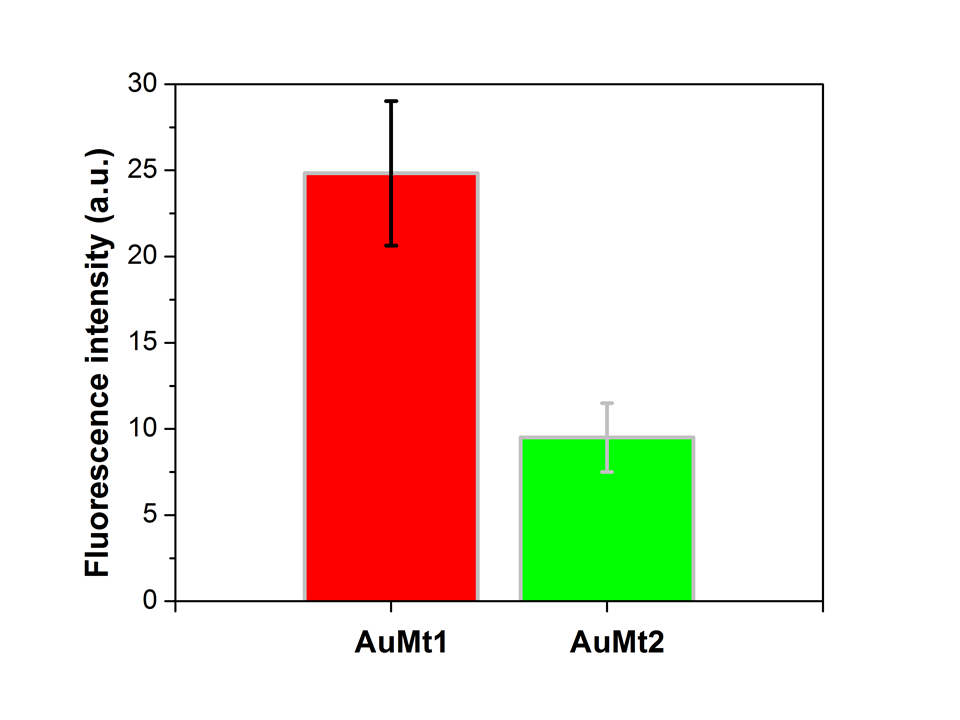
**

**Figure S7.** Fluorescence Intensity for AuMt1 and AuMt2 in HUVEC cells obtained for confocal microscopy.

**References**

1. Cruz MP, Andrade CM, Silva KO, de Souza EP, Yatsuda R, Marques LM, Clemente-Napimoga JT (2016) Antinoceptive and anti-inflammatory activities of the ethanolic extract, fractions and flavones isolated from Mimosa tenuiflora (Willd.) Poir (Leguminosae). PloS one 11(3):e0150839
2. Gerengi H, Schaefer K, Sahin HI (2012) Corrosion-inhibiting effect of Mimosa extract on brass-MM55 corrosion in 0.5 M H2SO4 acidic media. Journal of Industrial and Engineering Chemistry 18(6): 2204-2210
3. Souza RSOD, Albuquerque UPD, Monteiro JM, Amorim ELCD (2008) Jurema-Preta (Mimosa tenuiflora [Willd.] Poir.): a review of its traditional use, phytochemistry and pharmacology. Brazilian Archives of Biology and Technology 51(5): 937-947
4. Gaujac A, Aquino A, Navickiene S, De Andrade JB (2012) Determination of N, N-dimethyltryptamine in Mimosa tenuiflora inner barks by matrix solid-phase dispersion procedure and GC–MS. Journal of Chromatography B 881: 107-110
